# Triptonide-mediated PTGS2 Inhibition Induces Autophagic Cell Death to Suppress the Progression of Triple-negative Breast Cancer and Epithelial Ovarian Cancer

**DOI:** 10.7150/ijbs.127562

**Published:** 2026-01-30

**Authors:** Kunxiang Gong, Kai Song, Haotian Wang, Linye Li, Xiaomin Sun, Lingling Sun, Wenbo Hao, Zhe-Sheng Chen, Yinger Huang

**Affiliations:** 1 Scientific Research Center, First School of Clinical Medicine, The First Affiliated Hospital of Guangdong Pharmaceutical University, Guangzhou 510080, China.; 2 Guangzhou Institute of Pediatrics, Guangzhou Women and Children's Medical Center, Guangzhou Medical University, Guangzhou 510623, China.; 3 Institute of Antibody Engineering, School of Laboratory Medicine and Biotechnology, Southern Medical University, Guangzhou 510515, China.; 4 Department of Traditional Chinese Medicine, Zhujiang Hospital, Southern Medical University, Guangzhou 510280, China.; 5 Department of Oncology, Integrated Hospital of Traditional Chinese Medicine, Southern Medical University, Guangzhou 510315, China.; 6 Departments of Pharmaceutical Science, College of Pharmacy and Health Science, St John's University, Queens, NY 11439, USA.

**Keywords:** Triptonide, Prostaglandin G/H Synthase 2, Autophagic cell death, JAK/STAT3 signaling, Triple-negative breast cancer, Epithelial ovarian cancer

## Abstract

Triple-negative breast cancer (TNBC) and epithelial ovarian cancer (EOC) pose notable threats to the health of women. Given the poor prognosis associated with TNBC and EOC, new therapeutic agents must be explored urgently. Here, we identified triptonide (TN), a natural compound derived from the traditional Chinese herb *Tripterygium wilfordii*, as a potent antitumor agent. A series of functional assays showed that TN represses proliferation in TNBC and EOC cell lines, cell-derived xenograft, and patient-derived organoid models. Through molecular docking, molecular dynamics simulation, surface plasmon resonance, cell thermal shift assay, and drug affinity reaction target stability assays, we pinpointed PTGS2 as a direct target of TN. Mechanistically, TN binds to His-207 in PTGS2 and induces proteasome degradation of PTGS2 through recruiting E3 ubiquitin-protein ligase NEDD4. TN-induced PTGS2 downregulation leads to the inhibition of the JAK/STAT3/c-Myc signaling axis, resulting in suppression of tumor proliferation and the induction of autophagic cell death. In conclusion, our findings highlight TN as a promising candidate for TNBC and EOC treatment, acting through a novel mechanism involving targeted degradation of PTGS2 protein.

## Introduction

Triple-negative breast cancer (TNBC) and epithelial ovarian cancer (EOC), major malignancies that endanger women's health worldwide, are characterized by uniformly poor prognosis 1-3. Lacking specific therapeutic targets, TNBC and EOC depend on conventional chemotherapy, which is plagued by resistance 4-6. Recent advances in cancer research have introduced new hopes for targeted therapies 7, and poly ADP-ribose polymerase (PARP) inhibitors show efficacy in breast cancer susceptibility gene (*BRCA*)-mutant TNBC and EOC 8, 9 but are limited by eventual resistance 10, highlighting the demand for novel therapeutic strategies.

Prostaglandin-endoperoxide synthase 2 (PTGS2), also known as cyclooxygenase-2 (COX-2), is an enzyme expressed in various tissues that catalyzes the conversion of arachidonic acid to prostaglandins 11. Its products play critical roles in inflammatory signaling pathways 12. PTGS2 is frequently overexpressed in multiple tumor types, including colorectal cancer 13, breast cancer 14, and glioblastoma 15, where its expression correlates significantly with patient prognosis, suggesting its potential involvement in tumorigenesis, disease progression, and therapy resistance. These studies establish PTGS2 as a potential target for cancer treatment.

In TNBC, elevated PTGS2 expression promotes stemness and maintains tumor cell survival 16. Balamurugan et al. 17 reported that PTGS2 promotes breast cancer metastasis through stabilization of E-cadherin and β-catenin proteins. Similarly, in EOC, treatment with carboplatin induces PTGS2 upregulation, indicating its role in mediating platinum resistance 18. These findings collectively highlight PTGS2 as a promising therapeutic target for both TNBC and EOC, providing a rationale for further investigation into PTGS2-targeted therapies in these malignancies.

In the pursuit of targeted interventions, Traditional Chinese Medicine (TCM) has emerged as a rich reservoir of bioactive compounds with potential antitumor properties. For instance, baicalein, isolated from *Scutellaria baicalensis*, effectively suppresses the proliferation of lung cancer cells 19, while matrine, extracted from *Sophora flavescens*, exhibits notable anticancer activity against hepatocellular carcinoma and gastric cancer 20. Recently, the potential of TCM-derived bioactive compounds in the treatment of TNBC and EOC has gained considerable attention. However, the molecular targets and mechanisms of action for many of these compounds remain uncharacterized.

Triptonide (TN), a bioactive compound derived from the anti-inflammatory Chinese herbal medicine *Tripterygium wilfordii*, inhibits growth and induces cell death in multiple cancer cell types 21. TN exerts a marked antitumor effect, mainly by inducing ferroptosis through the SLC7A11/GPX4 axis 22 or by stabilizing B cell lymphoma 2 interacting mediator of cell death to enhance oxaliplatin-induced ferroptosis and apoptosis 23. Although a prior study has indicated that TN effectively inhibits metastasis in TNBC 24, the direct cytotoxicity and the underlying antitumor mechanism of TN in TNBC and EOC remain elusive. Given that PTGS2 is a canonical inflammatory mediator and TN is derived from a classic anti-inflammatory Chinese medicinal herb, we hypothesize that TN may exert antitumor effects by inhibiting PTGS2 expression. Accordingly, the present study aimed to elucidate the molecular mechanisms underlying TN-induced modulation of PTGS2 and its antitumor effects.

## Materials and Methods

### Ethical Statement

This study was approved by the Medical Ethics Committee of Guangzhou Women's and Children's Medical Center (Approval No.: 2025306A01). In accordance with the international ethical principles of the Declaration of Helsinki governing biomedical research, the collection and utilization of all human specimens were conducted following the procurement of written informed consent from each participant prior to tissue collection. The use of TNBC and EOC tumor tissue microarrays (TMAs) was authorized by the Ethics Committee of Outdo Biotech Company (Approval Nos.: SHYJS-CP-1807004 and SHYJS-CP-230702). The Experimental Animal Ethics Committee of Ruiye Model Animal (Guangzhou) Biotechnology Co., Ltd. approved animal experimental protocols (Approval No.: RYEth-20241201654).

### Cell culture and reagents

Human TNBC cell lines (SUM-159PT and MDA-MB-231), human EOC cell lines (SKOV3 and OVCAR3), normal human breast epithelial cell line (MCF10A), human colorectal carcinoma cell lines (HCT116 and RKO), and human embryonic kidney cell line (HEK-293T) were obtained from Procell Life Science & Technology (Wuhan, China). The IOSE-80 cell line, representing normal human ovarian surface epithelial cells, was acquired from iCell Bioscience Inc. (Shanghai, China). SUM-159PT, MDA-MB-231, HCT116, RKO, IOSE-80, and HEK-293T cells were cultured in Dulbecco's modified Eagle medium (DMEM, Gibco, USA) with 10% fetal bovine serum (FBS, Gibco) and 1% penicillin-streptomycin (Gibco). SKOV3 cells were cultured in McCoy's 5A medium (Gibco) with 10% FBS and 1% penicillin-streptomycin. OVCAR3 and MCF12A cell lines were maintained in their specific culture media (Cat# CM-0178 and Cat# CM-0791, Procell, respectively). All cell cultures in this study were kept under 20 passages, grown in a 37℃, 5% CO_2_ environment, and periodically monitored for Mycoplasma. HEK-293T cells were used to generate lentivirus with the lentiviral expression vector and packaging vectors. Triptonide (99.75% purity, Cat# HY-32736), dimethyl sulfoxide (DMSO) (Cat# HY-Y0320C), chloroquine (CHQ, Cat# HY-17589A), 3-Methyladenine (3-MA, Cat# HY-19312), cycloheximide (CHX, Cat# HY-12320), puromycin (Cat# HY-K1057), Pronase E (Cat# HY-114158A), TG101209 (Cat# HY-10410), ML115 (Cat# HY-111152), cisplatin (DDP, Cat# HY-17394), STAT3-IN-3 (Cat# HY-128588), and paclitaxel (PTX, Cat# HY-B0015) were purchased from MedChemExpress (Shanghai, China). Short hairpin RNA (shRNA) against *PTGS2*, small interfering RNA (siRNA) against neural precursor expressed, developmentally down-regulated protein 4 (NEDD4), siRNA against MDM2 proto-oncogene (MDM2), lentivirus plasmids expressing *PTGS2*, and a series of mutant *PTGS2* overexpressing plasmids were purchased from IGE Biotechnology (Guangzhou, China). NEDD4 overexpressing plasmid, wild type ubiquitin overexpressing plasmid, and a series of mutant ubiquitin overexpressing plasmids were purchased from TransSheep Bio Co. Ltd. (Shanghai, China). The RNA sequences are presented in Table S1.

### Cell viability and growth assay

Cells were plated into 96-well plates at 5,000 cells/well in complete growth medium and incubated overnight to facilitate attachment. Subsequently, they were treated with TN alone or in combination with other compounds for specified durations. Cell viability was assessed using the Cell Counting Kit-8 (CCK-8; Cat# C6005, NCM Biotech, China). Using DMSO as the vehicle control, results were calculated as background-subtracted relative absorbance values normalized to the vehicle control, based on triplicate experiments.

### Colony formation assay

For the colony formation assay, SUM-159PT, MDA-MB-231, and SKOV3 cells were plated in triplicate into 12-well plates at 5,000 cells per well. After substantial colony formation, the cells were treated with either 40 nM TN or vehicle control for 72 h. Following fixation with 4% paraformaldehyde and staining with crystal violet (each for 10 min), the plates were imaged to allow for colony quantification using ImageJ software.

### Tumor-sphere assay

For the tumor-sphere formation assay, 3,000 cells (SUM-159PT, MDA-MB-231, or SKOV3) were seeded in triplicate on ultra-low attachment plates in DMEM/F-12 medium containing 20 ng/mL EGF, 10 μg/mL insulin, 0.5 μg/mL hydrocortisone, and B27 supplement. After 72 h of cultivation, 40 nM TN was administered, and tumor spheres were assessed by counting and imaging after a 5-day treatment period.

### Cell cycle and apoptosis assays

Flow cytometry was employed to determine the cell cycle distribution and apoptosis rate in cells cultured under specific treatment conditions for the indicated durations. To assess cell cycle distribution and apoptosis, harvested cells were rinsed twice with PBS and subsequently stained using the cell cycle staining kit (Cat# KGA9101-100, KeyGen BioTECH, China) or Annexin V-based apoptosis detection reagents (either FITC conjugate, Cat# KGA1102-100, or APC conjugate, Cat# KGA1107-100, KeyGen BioTECH), respectively, following the manufacturer's protocols. Data were processed with ModFit LT software (v3.1, Verity Software House, USA) for quantification of cell cycle phases, while apoptosis rates were assessed using FlowJo software (vX.0.7, Tree Star Inc., USA).

### Cell migration by scratch assay

Following seeding into 6-well plates, cells were incubated in serum-free medium for 24 h to form a confluent monolayer, and linear scratches were then generated across the cell layer with a 200-μL pipette tip. Cells were then cultured in fresh serum-free medium containing either DMSO (control) or 40 nM TN for 24 h. To assess cell migration, images of the wound areas were captured at 0 h and 24 h post-scratch, and the migration rate was quantified by calculating the percentage of wound closure, defined as the reduction in the scratch wound area over 24 h relative to the initial area at 0 h.

### Immunoblotting and immunoprecipitation (IP)

Cells were lysed in western and IP cell lysis buffer (Cat# P70100, NCM Biotech) plus 1% protease inhibitor cocktail (Cat# P001, NCM Biotech). For immunoblotting, protein samples were processed through lysis in SDS-loading buffer, separation by SDS-PAGE, and electroblotting onto a polyvinylidene difluoride membrane (Cat# ISEQ00010, Millipore, USA). The membranes were then blocked with 5% skimmed milk for 2 h and incubated overnight at 4 °C with primary antibodies: β-actin (ACTB) (1:5000 dilution, Cat# 20536-1-AP, Proteintech, China), glyceraldehyde-3-phosphate dehydrogenase (GAPDH) (1:5000 dilution, Cat# 10494-1-AP, Proteintech), β-tubulin (1:5000 dilution, Cat# 10094-1-AP, Proteintech), cysteine-aspartic protease 3 (caspase-3) (1:1000 dilution, Cat# 66470-2-Ig, Proteintech), cleaved caspase-3 (1:1000 dilution, Cat# 341034, Zen BioScience), p62/SQSTM1 (p62) (1:5000 dilution, Cat# 18420-1-AP, Proteintech), LC3 (1:2000 dilution, Cat# 14600-1-AP, Proteintech), PTGS2 (1:1000 dilution, Cat# R23971, Zen BioScience, China), MDM2 (1:1000 dilution, Cat# 310329, Zen BioScience, China), HA tag (1:5000 dilution, Cat# 51064-2-AP, Proteintech), DYKDDDDK (Flag) tag (1:20000 dilution, Cat# 20543-1-AP, Proteintech), NEDD4 (1:2000 dilution, Cat# 21698-1-AP, Proteintech), phosphorylated extracellular signal-regulated kinase 1/2 (pERK1/2) (1:1000 dilution, Cat# 28733-1-AP, Proteintech), ERK1/2 (1:2000 dilution, Cat# 11257-1-AP, Proteintech), GTPase KRas (KRAS) (1:5000 dilution, Cat# 12063-1-AP, Proteintech), phosphorylated signal transducer and activator of transcription 3 (pSTAT3) (1:1000 dilution, Cat# AP0070, ABclonal, China), STAT3 (1:2000 dilution, Cat# 10253-2-AP, Proteintech), and c-Myc (1:2000 dilution, Cat# 10828-1-AP, Proteintech). After rinsing, membranes were exposed to HRP-conjugated secondary antibodies (Proteintech; anti-rabbit IgG, Cat# SA00001-2, 1:10000 and anti-mouse IgG, Cat# SA00001-1, 1:10000) for 1 h at room temperature and subsequently developed using the enhanced chemiluminescence substrate (Cat# P10060, NCM Biotech). For IP assay, an Anti-DYKDDDDK-tag FAST IP kit (Cat# EA-IP-K001, Elabscience Biotechnology Co., Ltd., China) was performed following the manufacturer's protocol. Data from 3 independent experiments are presented, with a representative western blot image shown, and the normalized band intensities were quantified using ImageJ software.

### Transmission electron microscopy (TEM)

After 48 h treatment with TN (40 nM), SUM-159PT, MDA-MB-231 and SKOV3 cells were collected, fixed with 2.5% glutaraldehyde at room temperature for 1 h, and subsequently incubated at 4℃ for 3 h prior to resin embedding, sectioning, and mounting onto copper grids. Images were captured on a Hitachi H-7650 instrument (Japan) with an operating voltage of 80 kV.

### Quantitative reverse transcription polymerase chain reaction (qRT-PCR) assay

We performed total RNA isolation from cells using the *AG RNAex Pro* RNA extraction reagent (Cat# AG21101, Accurate Biotechnology, China). For qRT-PCR analysis, complementary DNA (cDNA) was synthesized from the extracted RNA using the *Evo M-MLV* reverse transcription kit (Cat# AG11705, Accurate Biotechnology), which was then amplified employing the SYBR Green *Pro Taq* HS qPCR kit (Cat# AG11701, Accurate Biotechnology) on an Applied Biosystems 7500 Real-Time PCR System. Based on the GAPDH transcript as an internal control, all expression levels were normalized, and the corresponding primer sequences are provided in Table S2.

### Gene transfection

For siRNA transfection, cells were cultured until they reached 30-50% confluence and then transfected with 5 μL siRNA using Lipofectamine™ 3000 (Cat# L3000015, ThermoFisher Scientific, USA). For plasmid transfection, HEK-293T cells were grown at 70-80% confluence and transfected with indicated plasmids using Lipofectamine™ 3000. To establish stable cell lines, transfected cells were selected by treatment with puromycin for 5 days.

### Molecular docking and molecular dynamics (MD) simulations

Molecular docking simulations were carried out with AutoDock Vina v1.2.3 25, 26 to estimate binding affinities (kcal·mol⁻¹). Binding poses were visualized in 3D and 2D using PyMol v2.6 27 and Discovery Studio 2021 Client. The docked complexes were subjected to 100-ns MD simulations with GROMACS v2022.03 28; Gibbs free-energy profiles were derived via the integrated gmx_sham utility and xpm2txt.py script.

### Surface plasmon resonance (SPR) assay

SPR analysis was employed to characterize the binding interaction between TN and PTGS2 protein. Using a running buffer containing 5% DMSO, TN was serially diluted to various concentrations. Recombinant PTGS2 protein was immobilized as the ligand on a CM5 sensor chip (GE Healthcare, Marlborough, MA, USA). The binding assays were conducted at 25°C on a Biacore T200 instrument (GE Healthcare), with subsequent analysis of the resulting binding sensorgrams carried out using the Biacore T200 Control Software (v. 2.0, GE Healthcare) to determine the binding kinetics.

### Cell thermal shift assay (CETSA) and drug affinity responsive target stability (DARTs)

Protein lysates were prepared by lysing cells in ice-cold lysis buffer supplemented with 1% protease inhibitor, followed by centrifugation. The lysates were then incubated with either 100 μM TN or an equivalent volume of DMSO for 1 h at room temperature. Following a described CETSA procedure 29, the samples were subjected to a gradient of increasing temperatures for 3 min each and then cooled at 4ºC for 3 min. After centrifugation for 20 min (10,000 ×*g*, 4ºC), the soluble supernatant was subjected to western blot. For the DARTs assay, Pronase E (2%) was added to both the TN- and DMSO-treated lysates and incubated at room temperature for 30 min. After centrifugation at 10,000 ×*g* for 20 min at 4ºC, the resulting supernatants were analyzed by western blot.

### Bioinformatics analysis

Potential protein targets of TN were identified via the SwissTargetPrediction database (http://www.swisstargetprediction.ch/). Gene expression for *PTGS2*, *F2RL1*, *KCNA3*, *NR3C1*, and *AR* were obtained from The Cancer Genome Atlas (TCGA) utilizing the UCSC Xena browser 30. Survival analysis based on *PTGS2* expression in ovarian cancer and basal-like breast cancer was performed with the Kaplan-Meier plotter tool 31. Furthermore, the UbiBrowser 2.0 database 32 was employed to predict E3 ligases that potentially mediate the ubiquitination of PTGS2 protein.

### RNA sequencing (RNA-seq) analysis

Total RNA (≥5 μg per sample) was isolated from SUM-159PT cells transfected with either PTGS2-targeting or control shRNA (n = 3/group). RNA sequencing and primary data analysis were contracted to Novogene Co., Ltd. (Beijing, China). Functional annotation and pathway enrichment were subsequently conducted using Gene Set Enrichment Analysis (GSEA).

### Mice xenograft model

Female BALB/c nude mice (4-5 weeks) were obtained from the Experimental Animal Center of Southern Medical University, followed by being housed in autoclaved, ventilated cages with access to autoclaved water. 2 × 10^6^ MDA-MB-231 or 5 × 10^6^ SKOV3 cells were injected subcutaneously. When the average tumor volume reached approximately 90 mm³, designated as day 0, the tumor-bearing mice were randomly allocated to three groups: (1) vehicle control group, (2) TN treatment group (8 mg/kg/qd), and (3) positive control group receiving PTX (10 mg/kg every three days) 33 (n = 6/group). The vehicle group was administered a solution consisting of 5% DMSO, 45% PEG 400, 5% Tween-80, and 45% saline (all volume/volume, v/v). All administration methods are intraperitoneal injection. Treatments were discontinued after 10 days. Tumor growth was evaluated through caliper measurements at designated time points, and tumor volume was calculated according to the formula: *length × width^2^*/2; tumors were subsequently photographed and weighed to complete the assessment.

### Immunohistochemistry (IHC) and hematoxylin-eosin (H&E) staining

IHC and H&E staining were conducted using previously described methods 34. For IHC analysis, tissue sections were subjected to antigen retrieval and blocking, then incubated overnight at 4°C with specific primary antibodies against PTGS2 (1:200 dilution, Cat# 12375-1-AP, Proteintech), Ki-67 (1:4000 dilution, Cat# 27309-1-AP, Proteintech), p62 (1:200 dilution, Cat# 18420-1-AP, Proteintech), c-Myc (1:500 dilution, Cat# 10828-1-AP, Proteintech), and pSTAT3 (1:100 dilution, Cat# 9145, Cell Signaling Technology). Subsequently, the sections were washed three times in PBS and incubated with a secondary antibody for 2 h at 37°C, and signal detection was achieved using diaminobenzidine. PTGS2 expression in TMAs was scored according to established standards 35. To perform H&E staining, sections were first incubated with hematoxylin for 5 min and then subjected to eosin treatment for the same duration.

### Patient-derived organoid (PDO) model construction and culture

Fresh tumor tissue specimens from patients with EOC were minced into small fragments, rinsed with PBS, and digested with type IV collagenase (Cat# 17104019, ThermoFisher Scientific) at 37 °C for 1 h. The cell suspension was sequentially filtered through a 100 μm strainer, centrifuged, and subjected to erythrocyte lysis. The isolated cells were resuspended in Advanced DMEM/F12 medium supplemented with growth factor-reduced Matrigel (Cat# CB-40230, Corning, USA); the obtained cell-Matrigel suspension was overlaid with a specialized organoid culture medium (Cat# CP-11206, Guangzhou Orgen Biotechnology Co., Ltd., China) and maintained at 37 °C under 5% CO_2_, with regular medium replenishment performed every 2-3 days. To assess the proliferative status of the resulting EOC PODs, cell viability was measured using the Cell Counting-Lite 3D Luminescent Cell Viability Assay (Cat# DD1102-01, Vazyme, China), with three independent replicate wells set up for each group to minimize experimental variation.

### Statistical analysis

Data derived from independent experiments are expressed as mean ± SD, with all quantitative analyses implemented using GraphPad Prism 6.0. The normality of data was assessed using the Kolmogorov-Smirnov test prior to applying the two-tailed Student's t-test for comparisons between two groups. For comparisons across three or more groups, one-way analysis of variance (ANOVA) followed by Dunnett's post hoc test was used. Two-way ANOVA with Dunnett's post hoc test was employed to analyze cell growth curves, IC_50_ curves, and PTGS2 protein expression curves, whereas two-way ANOVA with Šídák's post hoc test was used for tumor volume growth curves. The correlation between PTGS2 IHC scores and the Ki-67 index or tumor burden was evaluated by Spearman's correlation analysis. Significance was set at *P* < 0.05 (*, **, *** for *P* < 0.05, 0.01, 0.001; ns, not significant).

## Results

### Triptonide induces autophagic cell death in TNBC and EOC cells

**Figure 1:**
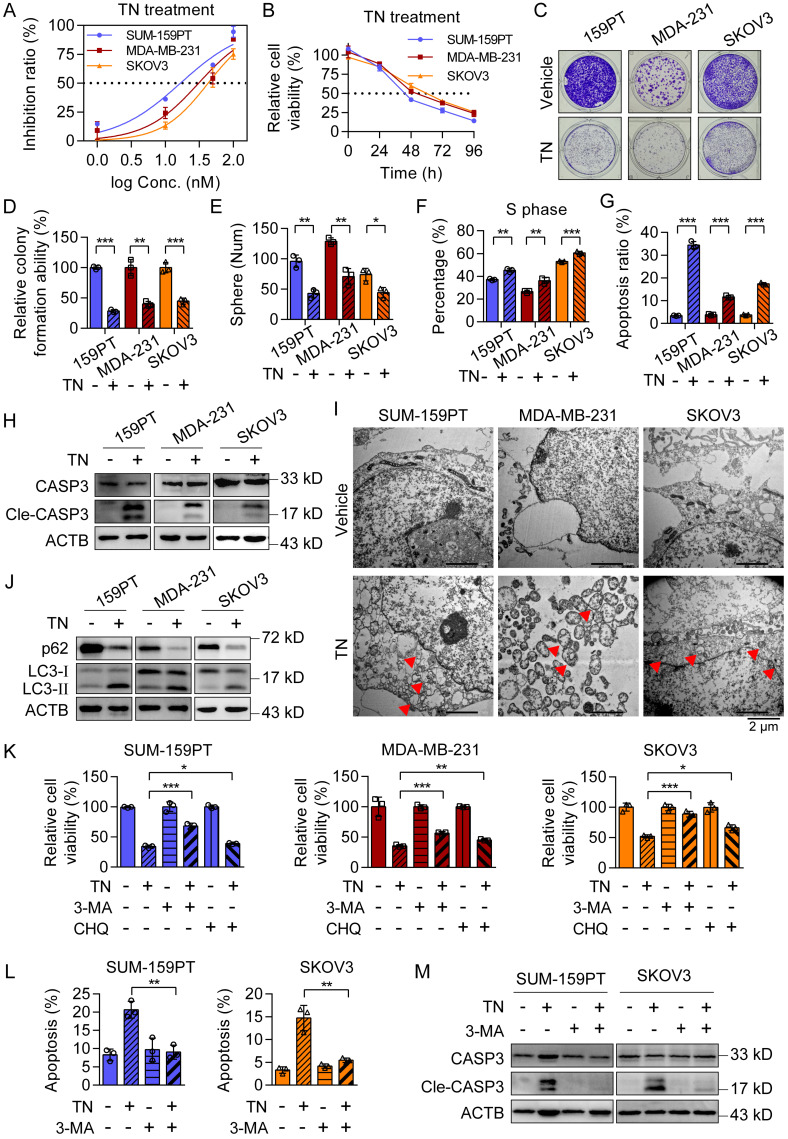
** Triptonide significantly inhibits the proliferation and promotes apoptosis of TNBC and EOC cells. (A)** Dose-response curves of TN in SUM-159PT, MDA-MB-231, and SKOV3 cells after 72 h treatment. **(B)** Growth curves of the three cell lines treated with 40 nM TN at the indicated time points. **(C-D)** Colony formation ability of the cells after treatment with 40 nM TN for 72 h. Representative images (C) and quantitative analysis (D) are shown. **(E)** Tumorsphere formation after 5 days of treatment with 40 nM TN. **(F)** Quantification of cell cycle percentage using flow cytometry analyses after 24 h of treatment with 40 nM TN. **(G)** Apoptosis was quantified using flow cytometry after 48 h exposure to 40 nM TN. **(H)** Western blot analysis of cleaved caspase-3 after 72 h treatment of the cells with 40 nM TN.** (I)** TEM images showing autophagic vacuoles (*red arrows*) in the cell lines after 24 h treatment with 40 nM TN. **(J)** Western blot analysis of p62, LC3-I, and LC3-II after 48 h treatment with 40 nM TN. **(K)** Viability of the cells pretreated with 3-MA (5 mM, 6 h) or CHQ (25 μM, 6 h), and then exposed to TN (40 nM, 48 h), as measured using the CCK-8 assay. **(L)** Apoptosis of SUM-159PT and SKOV3 cells pretreated with 3-MA (5 mM, 6 h) and then exposed to TN (40 nM, 48 h) was quantified using flow cytometry. **(M)** Cleaved caspase-3 expression levels in SUM-159PT and SKOV3 cells pretreated with 5 mM 3-MA for 6 h and subsequently exposed to 40 nM TN for 48 h were determined by western blot. All data are expressed as mean ± SD, with statistical significance assessed via Student's t-test; *P < 0.05, **P < 0.01, ***P < 0.001.

To evaluate the cytotoxic effect of TN on TNBC and EOC cells, we incubated two typical TNBC cell lines (SUM-159PT and MDA-MB-231) and one EOC cell line (SKOV3) with a gradient of TN concentrations for 72 h. The results showed that TN exposure significantly reduced cell viability in a dose-dependent manner across all cell lines (Fig. 1A). Subsequent treatment with 40 nM TN for 4 days revealed a time-dependent decline in cell viability, as measured using CCK-8 assay (Fig. 1B). Nonetheless, TN exhibits a significantly reduced growth-inhibitory effect on normal breast epithelial MCF10A cells relative to TNBC cells, as well as on normal human ovarian surface epithelial IOSE-80 cells compared to EOC cells (Fig. S1A-B). Notably, at a concentration comparable to that of the first-line clinical drug PTX, TN also exerted potent toxicity against TNBC and EOC cells (Fig. S1C), highlighting its potential for clinical translation. Furthermore, TN effectively suppressed clonogenic ability (Fig. 1C-D) and tumorsphere-forming capacity (Fig. 1E and Fig. S1D) in both TNBC and EOC cells. The flow cytometry analysis demonstrated a significant increase in the proportion of cells in the S phase following TN treatment (Fig. 1F and Fig. S1E), suggesting S-phase cell cycle arrest. To determine whether this growth inhibition was associated with apoptosis, we performed flow cytometry-based apoptosis assays and found that TN induced significant apoptosis in TNBC and EOC cells compared to controls (Fig. 1G and Fig. S1F). Consistent with these findings, western blot analysis confirmed elevated levels of cleaved caspase-3 in TN-treated groups (Fig. 1H). Electron microscopy analysis revealed the formation of autophagic vacuoles in all three cell lines following TN treatment (Fig. 1I). To further verify whether TN induces autophagy in TNBC and EOC cells, we treated SUM-159PT, MDA-MB-231, and SKOV3 cells with 40 nM TN for 48 h. Western blot analysis showed a marked decrease in the expression of the autophagy-related protein p62 and a significant increase in LC3-II levels in all three cell lines (Fig. 1J). Moreover, the qPCR analysis results indicated that the mRNA expression of several autophagy-related genes was upregulated to varying degrees in both MDA-MB-231 and SKOV3 cells after TN treatment (Fig. S1G). As autophagy represents a critical cellular process for maintaining homeostasis and promoting cell survival, we sought to determine whether its activation in response to TN represents a protective mechanism or contributes to cell death. To address this, rescue experiments were conducted using the autophagy inhibitors 3-MA (5 mM) and CHQ (25 μM). Cells were pretreated with either inhibitor for 6 h before the addition of 40 nM TN. CCK-8 assays showed that inhibition of autophagy partially attenuated TN-induced cell death (Fig. 1K). Furthermore, both flow cytometry and western blot analyses revealed that pretreatment with 3-MA significantly reduced TN-mediated apoptosis in TNBC and EOC cells (Fig. 1L-M and Fig. S1H). Overall, these findings indicate that TN effectively inhibits the proliferation of TNBC and EOC cells and induces autophagic cell death.

### PTGS2 is a direct target of triptonide and contributes to TNBC and EOC malignancy

**Figure 2:**
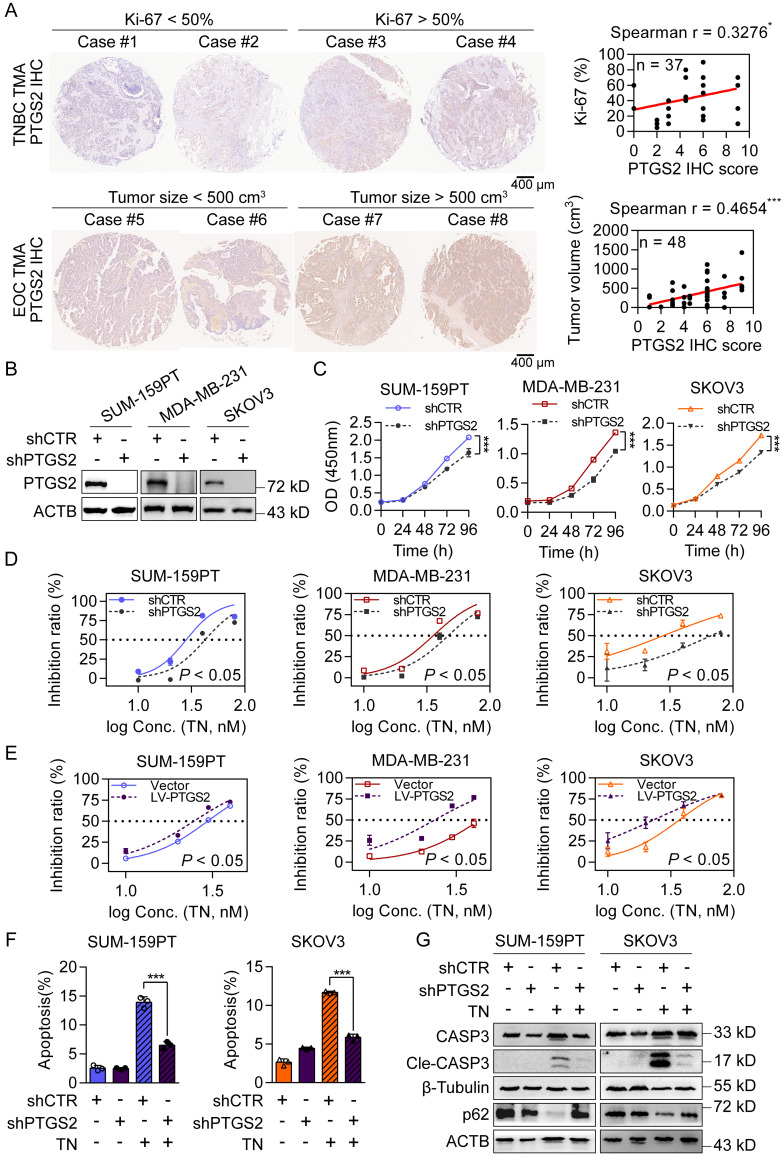
** Triptonide targets the PTGS2 protein, which contributes to the progression of TNBC and EOC. (A)** Representative images (*left*) and correlation analysis (*right*) of PTGS2 IHC score vs. Ki-67 index in TNBC tumor tissues (*top*), and PTGS2 IHC score vs. tumor volume in patients with EOC (*bottom*). **(B)** Western blot analysis of PTGS2 expression in shRNA control or *PTGS2*-targeting shRNA-transduced SUM-159PT, MDA-MB-231. and SKOV3 cells. **(C)** CCK-8 assay of proliferation in shRNA control and *PTGS2*-knockdown SUM-159PT, MDA-MB-231, and SKOV3 cells. **(D)** TN IC_50_ curves for *PTGS2*-knockdown and shRNA control SUM-159PT, MDA-MB-231, and SKOV3 cells. **(E)** TN IC_50_ curves for vector control vs. *PTGS2*-overexpressing SUM-159PT, MDA-MB-231, and SKOV3 cells. **(F)** Flow cytometry-evaluated apoptosis of *PTGS2*-knockdown and shRNA control SUM-159PT, SKOV3 cells (20 nM TN, 48 h). **(G)** Assessment of apoptosis- and autophagy-related proteins via western blot in PTGS2-knockdown and shRNA control SUM-159PT, SKOV3 cells (40 nM TN, 48 h). Data are reported as mean ± SD, with statistical significance evaluated via Spearman's correlation analysis, two-way ANOVA, or Student's t-test; *P < 0.05, ***P < 0.001.

To further investigate the antitumor mechanisms of TN, we utilized the SwissTargetPrediction database to identify potential protein targets. PTGS2 emerged as the top-ranked candidate (Fig. S2A). Notably, among the top five candidates, *PTGS2* mRNA expression was highest in basal-like breast cancer tissues (Fig. S2B). Analysis of clinical data revealed that patients with TNBC and EOC with high *PTGS2* expression had significantly shorter survival (Fig. S2C). Additionally, tissue microarray analyses demonstrated that in TNBC specimens, PTGS2 protein expression was positively correlated with the proliferation marker Ki-67, whereas in EOC specimens, it correlated with increased tumor burden (Fig. 2A). To assess the functional role of PTGS2, we constructed stable *PTGS2*-knockdown and PTGS2-overexpressing clones in SUM-159PT, MDA-MB-231 and SKOV3 cells (Fig. 2B and Fig. S2D). CCK-8 assays showed that PTGS2 suppressed cell proliferation, whereas the overexpression of PTGS2 exerted the opposite effect (Fig. 2C and Fig. S2E), indicating PTGS2 promotes tumorigenesis in TNBC and EOC. Crucially, PTGS2 knockdown increased the IC_50_ values of TN (Fig. 2D), conferring partial resistance to its antitumor effects, while PTGS2 overexpression sensitized cells to TN, lowering IC_50_ values (Fig. 2E). Flow cytometry analysis further revealed that PTGS2 knockdown attenuated TN-induced apoptosis (Fig. 2F and Fig. S2F), implying reduced cellular sensitivity to TN-mediated cytotoxicity. Moreover, the combination of PTGS2 knockdown with subsequent TN treatment led to decreased levels of autophagic cell death, as evidenced by diminished expression of cleaved caspase-3 and elevated levels of p62 protein in both TNBC and EOC cells (Fig. 2G). Furthermore, we overexpressed PTGS2 in OVCAR3, an EOC cell line with low endogenous PTGS2 expression (Fig. S2G). The IC_50_ curve analysis showed that PTGS2 overexpression sensitized OVCAR3 cells to TN (Fig. S2H). Additionally, we found that TN effectively inhibited the migratory capacity of TNBC and EOC cells (Fig. S2I). As elevated PTGS2 expression has also been shown to enhance the metastatic potential of TNBC cells 40, we assessed the effect of TN on the migratory capacity of MDA-MB-231 cells following *PTGS2* knockdown. Our data demonstrated that *PTGS2* knockdown attenuated the inhibitory effect of TN on TNBC cell migration (Fig. S2J). These results suggest that PTGS2 serves as a critical target for TN-induced autophagic cell death and its antitumor efficacy.

### Binding of triptonide to His-207 of the PTGS2 protein is essential for its anticancer bioactivity

**Figure 3:**
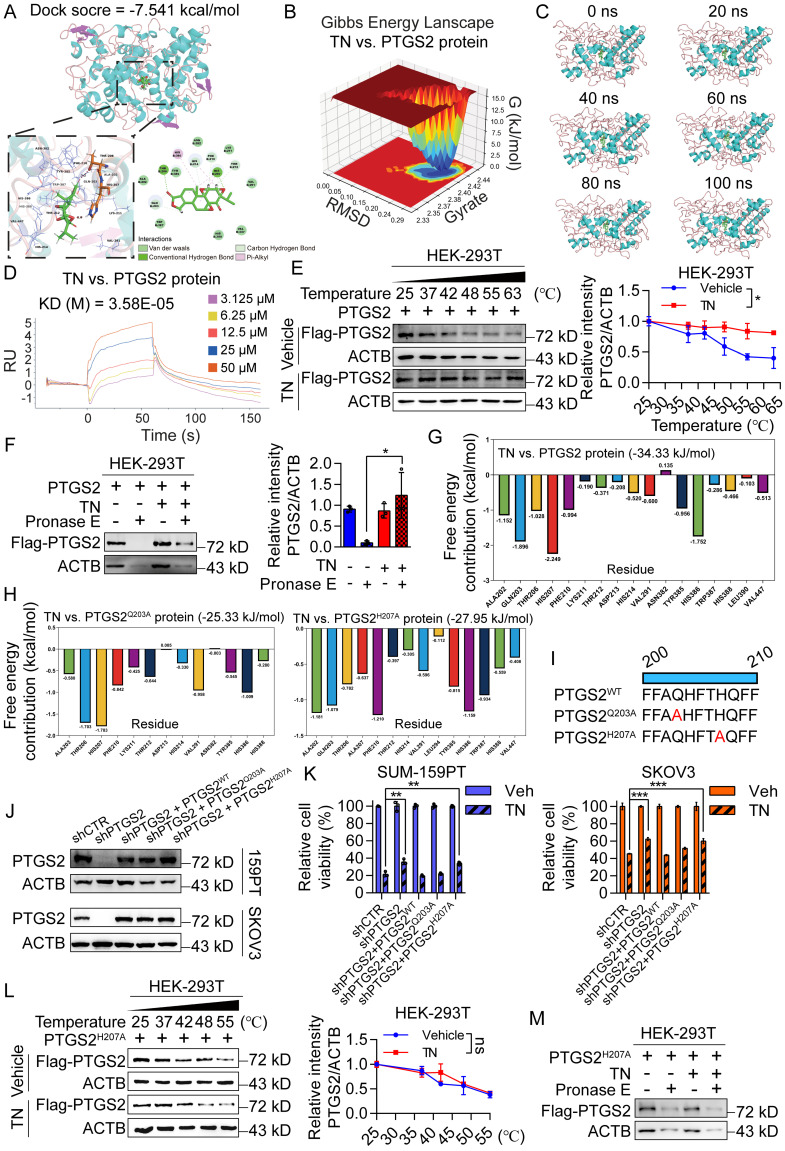
** Triptonide exerts anticancer bioactivity by directly binding to His-207 of the PTGS2 protein. (A)** Molecular docking analysis of PTGS2 and TN. **(B)** Three-dimensional Gibbs free energy landscape of the PTGS2-TN binding system. **(C)** Analysis of the 100 ns molecular dynamics simulation trajectory of the PTGS2-TN complex. **(D)** Binding affinity between TN and PTGS2 protein measured by SPR analysis. **(E-F)** CETSA (E) and DARTs (F) assays were used to detect the binding between TN and PTGS2 in PTGS2-overexpressing HEK-293T cells. Representative blot (*left*) and quantitative analysis (*right*) are shown. **(G)** Free energy distribution at individual binding sites following TN binding to wild-type PTGS2. **(H)** Free energy distribution at binding sites after TN binding to PTGS2^Q203A^ and PTGS2^H207A^ mutants. **(I)** Schematic representation of wild-type PTGS2 and catalytic-site mutants. **(J)** Western blot analysis of PTGS2 expression in wild-type/mutant PTGS2 cell lines. **(K)** Viability of cells expressing wild-type or mutant PTGS2 after treatment with 40 nM TN or DMSO for 72 h. **(L)** CETSA analysis of TN binding to PTGS2^H207A^ mutant. Representative blot (*left*) and quantitative analysis (*right*) are shown. **(M)** Western blot analysis of degradation of PTGS2^H207A^ mutant protein in TN-treated cell lysate under pronase E treatment. Data are expressed as mean ± SD, with statistical significance evaluated via two-way ANOVA, or Student's t-test; *P < 0.05, **P < 0.01, ***P < 0.001, ns, no significance.

Further, we performed molecular docking simulations to predict the potential interaction between TN and the PTGS2 protein. The results demonstrated that TN binds to PTGS2 with a docking score of -7.541 kcal/mol, suggesting high-affinity binding (Fig. 3A). To further characterize this interaction, we conducted MD simulations. The three-dimensional Gibbs free energy landscape revealed that the binding of TN to PTGS2 formed a single, well-defined low-energy cluster (Fig. 3B), indicating a stable binding conformation. Subsequent MD stability analyses confirmed that TN can form stable complexes with PTGS2 (Fig. 3C and Fig. S3A-B). To experimentally validate the binding affinity, we expressed and purified recombinant PTGS2 protein (Fig. S3C). SPR analysis indicated that TN binds to PTGS2 with high affinity (Fig. 3D). Furthermore, CETSA and DARTs experiments consistently demonstrated effective direct binding between TN and PTGS2 in cellular contexts (Fig. 3E-F). Additional molecular docking analyses indicated that the total binding free energy for the TN-PTGS2 interaction was -34.33 kJ/mol. Among the residues, histidine 207 (H207) and glutamine 203 (Q203) contributed most significantly to the binding energy (Fig. 3G). To investigate whether these residues are critical for binding stability, we generated alanine substitutions (H207A and Q203A) and performed 100 ns MD simulations. Results revealed that both mutations impaired the stability of TN-PTGS2 binding (Fig. S3D-E). Moreover, compared to the wild-type system, both mutants exhibited decreased binding free energy (Fig. 3H), indicating that H207 and Q203 play crucial roles in maintaining complex stability. To further examine which residue is essential for the anticancer bioactivity of TN, we constructed PTGS2 mutant plasmids carrying Q203A (PTGS2^Q203A^) and H207A (PTGS2^H207A^) mutations (Fig. 3I). These mutants, along with wild-type PTGS2 (PTGS2^WT^), were re-expressed in *PTGS2*-knockdown SUM-159PT and SKOV3 cell clones (Fig. 3J). Notably, in both cell lines, the diminished sensitivity to TN observed in *PTGS2*-knockdown cells was markedly rescued by re-expression of either wild-type PTGS2 or the PTGS2^Q203A^ mutant; however, not by the PTGS2^H207A^ mutant (Fig. 3K). Furthermore, we expressed the PTGS2^H207A^ protein in HEK-293T cells and conducted CETSA and DARTs assays. We found that mutation at the His-207 site of PTGS2 abrogated the effective binding between TN and the PTGS2 protein (Fig. 3L-M and Fig. S3F). Collectively, these results demonstrate that TN inhibits the progression of TNBC and EOC by targeting PTGS2, and that His-207 in PTGS2 is essential for the binding of TN to the PTGS2 protein.

### Triptonide serves as a PTGS2 degrader by recruiting E3 ligase NEDD4 in ubiquitin-proteasome system

**Figure 4:**
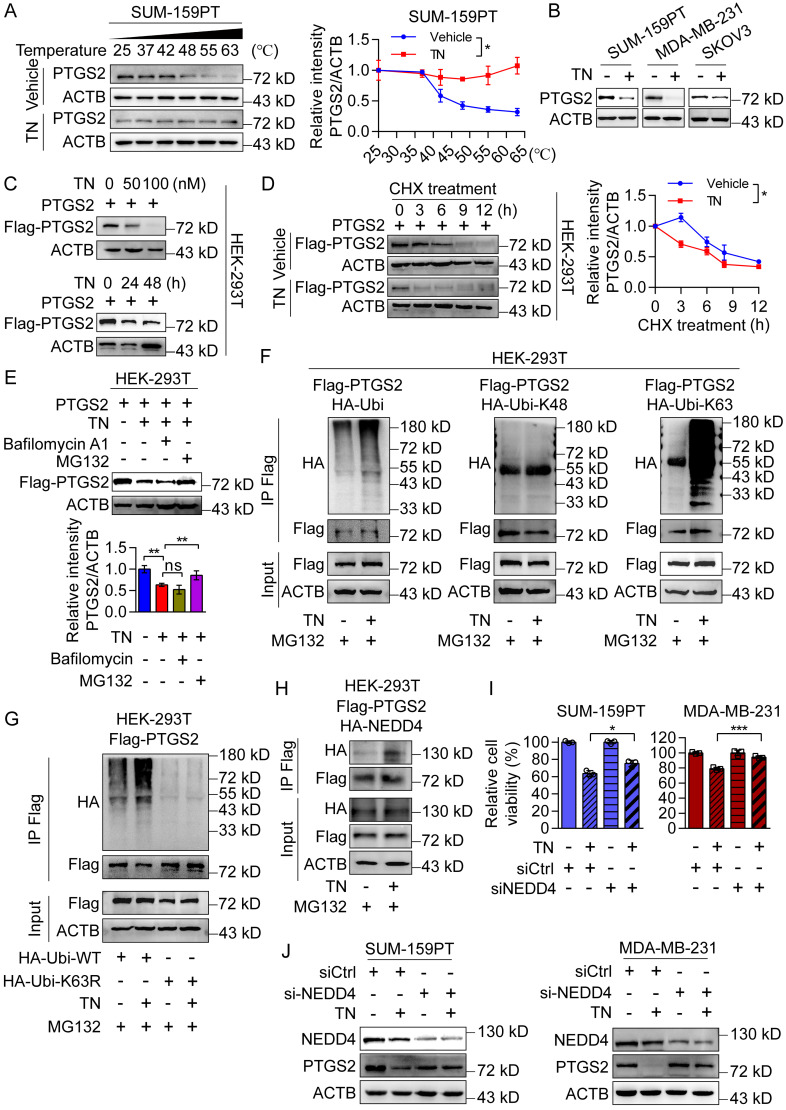
** Triptonide promotes PTGS2 degradation via the ubiquitin-proteasome system by recruiting the E3 ligase NEDD4. (A)** CETSA assay of TN-treated SUM-159PT cell lysate; DMSO-treated cell lysate was used as a negative control, and representative blot (*left*) and quantitative analysis (*right*) are shown. **(B)** PTGS2 protein expression in SUM-159PT, MDA-MB-231, and SKOV3 cells treated with 40 nM TN for 48 h was assessed by western blot. **(C)** The inhibitory effect of TN on PTGS2 protein expression was detected by western blot. **(D)** Effect of TN on PTGS2 expression in the presence of time-course CHX (100 μg/mL) treatment assessed by western blot. Representative blot (*left*) and quantitative analysis (*right*) are shown. **(E)** Effect of TN on PTGS2 expression following pretreatment with bafilomycin A1 (5 μM) or MG132 (10 μM), evaluated using western blot. Representative blot (*upper*) and quantitative analysis (*bottom*) are shown. **(F)** HEK-293T cells co-expressing PTGS2 and ubiquitin (or K48/K63 ubiquitin mutants) were pretreated with MG132 (10 μM, 6 h), followed TN (50 nM, 24 h) treatment, and the ubiquitination pattern of PTGS2 protein was assessed by co-IP assay. **(G)** Co-IP assay to examine the effect of TN on PTGS2 ubiquitination following K63 site mutation in ubiquitin. **(H)** HEK-293T cells co-expressing PTGS2 and NEDD4 were pretreated with MG132 and exposed to TN for 24 h. Co-IP assay was performed to detect the binding of PTGS2 and NEDD4. **(I)** CCK-8 detection of cell viability in TN-treated NEDD4-knockdown TNBC cells. Cell viability was measured using the CCK8 assay. **(J)** NEDD4-silenced SUM-159PT and MDA-MB-231 cells were subjected to 40 nM TN treatment for 48 h, with PTGS2 expression analyzed by western blot. Data are expressed as mean ± SD, with statistical significance assessed by two-way ANOVA, or Student's t-test; *P < 0.05, **P < 0.01, ***P < 0.001.

Subsequently, we confirmed the direct binding of TN to PTGS2 in SUM-159PT and MDA-MB-231 cells (Fig. 4A and Fig. S4A) and observed that TN effectively inhibited PTGS2 protein expression without altering *PTGS2* mRNA levels (Fig. 4B and Fig. S4B), suggesting that TN regulates PTGS2 expression predominantly at a post-transcriptional level. To further investigate the impact of TN binding on PTGS2 protein expression, we exogenously expressed PTGS2 in HEK-293T cells and treated them with TN. Western blot analysis revealed that TN significantly reduced PTGS2 protein levels in a concentration- and time-dependent manner (Fig. 4C and Fig. S4C). Moreover, to determine whether TN influences PTGS2 protein stability, we inhibited protein synthesis using CHX and subsequently monitored PTGS2 protein levels. Compared with cells receiving vehicle treatment, TN treatment notably reduced the half-life of the PTGS2 protein (Fig. 4D), indicating that TN binding compromises PTGS2 protein stability and promotes its degradation. Further, pretreatment with Bafilomycin A1 (a lysosome pathway inhibitor) and MG132 (a ubiquitin proteasome pathway inhibitor) indicated that TN predominantly degraded PTGS2 through the proteasome system rather than the lysosomal pathway (Fig. 4E). To determine whether TN-induced PTGS2 degradation triggers autophagic cell death, we performed rescue experiments using the proteasome inhibitor MG132. The CCK-8 assay results showed that MG132 pretreatment alleviated TN-induced cytotoxicity (Fig. S4D). The western blot analysis further revealed that MG132 restored PTGS2 levels and downregulated p62 expression (Fig. S4E), indicating that TN induces autophagic cell death via PTGS2 degradation. Next, our Co-IP results showed that TN significantly promotes the ubiquitination modification of PTGS2 protein, particularly, this ubiquitination modification was predominantly mediated by K63-linked ubiquitin chain assembly rather than K48-linked chain formation (Fig. 4F-G). Furthermore, we expressed the PTGS2^H207A^ mutant in HEK-293T cells and treated them with TN. The results showed that after the His-207 site mutation, TN could not effectively promote the ubiquitination of PTGS2 protein (Fig. S4F), and the inhibitory effect of TN on PTGS2 protein was lost (Fig. S4G). Previous literature suggests that E3 ubiquitin ligase NEDD4 is a key ligase that promotes PTGS2 ubiquitination. The database prediction also indicated that NEDD4 promotes the most reliable ubiquitination degradation of PTGS2 (Fig. S4H). The Co-IP results also showed that TN treatment promoted the binding of PTGS2 and NEDD4 (Fig. 4H).

To determine whether the promotion of PTGS2 ubiquitination degradation by TN requires the involvement of NEDD4, we knocked down NEDD4 in SUM-159PT and MDA-MB-231 cells and treated them with the same concentration of TN. The CCK-8 assay showed that knocking down NEDD4 alleviated the killing effect of TN on tumor cells (Fig. 4I), and the western blot results also revealed that compared with the control group, knocking down NEDD4 weakened the inhibitory effect of TN on PTGS2 protein (Fig. 4J), indicating that E3 ligase NEDD4 is involved in the ubiquitination degradation process of PTGS2 by TN. Considering that MDM2 also mediates PTGS2 ubiquitination and degradation, we examined its role in TN-induced cytotoxicity by knocking it down in SUM-159PT cells (Fig. S4I). The CCK-8 assay results showed that *MDM2* knockdown did not alter the cytotoxic effect of TN (Fig. S4J), indicating that MDM2 is not involved in TN-mediated PTGS2 degradation. Thus, these data suggest that the binding of TN to PTGS2 protein promotes the interaction between NEDD4 and PTGS2, subsequently enhancing proteasomal degradation of PTGS2.

### Triptonide exerts its tumor-killing effect by downregulating the JAK/STAT3 signaling pathway

**Figure 5:**
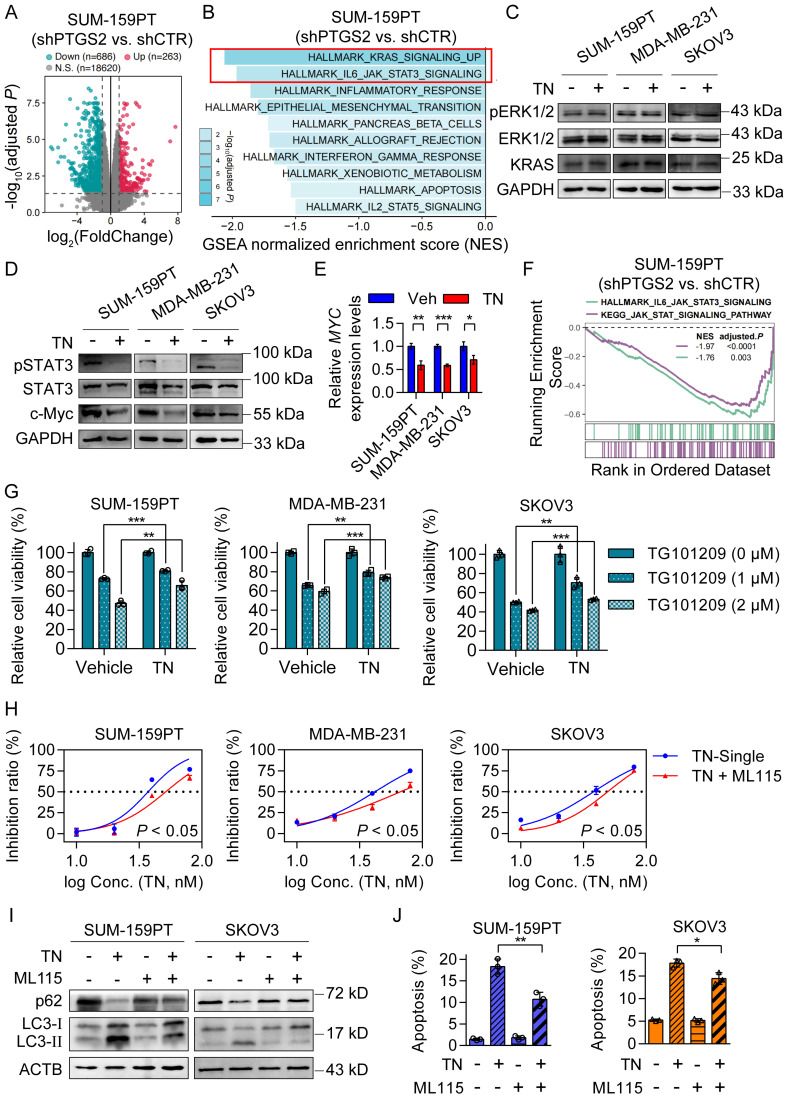
** Triptonide induces autophagic cell death by downregulating the JAK/STAT3 signaling pathway. (A)** Differential gene expression analysis (|log2FC| > 1, adjusted *P* < 0.05) in shRNA control and *PTGS2*-knockdown SUM-159PT cells. **(B)** GSEA of downregulated genes in shRNA control and *PTGS2*-knockdown SUM-159PT cells. **(C-D)** Western blot analysis of KRAS/ERK signaling (C) and STAT3/c-Myc signaling (D) in SUM-159PT, MDA-MB-231, and SKOV3 cells after TN (40 nM, 72 h) treatment. **(E)** MYC mRNA quantification by qPCR in TN-exposed SUM-159PT, MDA-MB-231, and SKOV3 cells. **(F)** GSEA of HALLMARK_IL6_JAK_STAT3_ SIGNALING and KEGG_JAK_STAT_SIGNALING pathways in shRNA control and *PTGS2*-knockdown SUM-159PT cells. **(G)** After TN pretreatment (40 nM, 12 h), SUM-159PT, MDA-MB-231, and SKOV3 cells were exposed to TG101209 (1 or 2 µM, 24 h), and viability was measured using the CCK-8 method. **(H)** Cytotoxic effect of TN in SUM-159PT, MDA-MB-231, and SKOV3 cells after addition of ML115 (10 µM). **(I)** Changes in p62 and LC3-II protein levels in SUM-159PT and SKOV3 cells after TN and ML115 treatment, detected by western blot. **(J)** Apoptosis in SUM-159PT and SKOV3 cells after TN and ML115 treatment, assessed by flow cytometry. Data are expressed as mean ± SD, with statistical significance assessed by two-way ANOVA, or Student's t-test; *P < 0.05, **P < 0.01, ***P < 0.001.

To investigate the downstream signaling pathways by which TN suppresses PTGS2 protein expression and mediates its antitumor effects, we conducted RNA-seq using *PTGS2*-knockdown and control SUM-159PT cells. The RNA-seq result showed that 263 genes were upregulated and 686 genes were downregulated upon PTGS2 knockdown (Fig. 5A). Subsequent Hallmark GSEA analysis indicated that the top two downregulated pathways in SUM-159PT cells after PTGS2 knockdown were the KRAS signaling pathway and the IL6-JAK-STAT3 pathway (Fig. 5B). Previous studies have indicated that inhibition of the KRAS/ERK signaling axis 36 and downregulation of the STAT3/c-Myc pathway 37 can both trigger autophagic cell death. To investigate which signaling axis mediates the cytotoxic effects of TN on TNBC and EOC cells following PTGS2 suppression, we treated SUM-159PT, MDA-MB-231, and SKOV3 cells with 40 nM TN for 72 h and analyzed protein expression changes via western blot analysis. The results demonstrated that the expression of pSTAT3 and c-Myc proteins was significantly reduced, instead of pERK1/2 and KRAS protein (Fig. 5C-D and Fig. S5A-B). Simultaneously, qPCR results showed that *MYC* mRNA expression was also suppressed by TN treatment (Fig. 5E). GSEA based on KEGG pathways further revealed that knockdown of PTGS2 led to significant downregulation of the JAK/STAT signaling pathway (Fig. 5F and Fig. S5C). The western blot analysis revealed that silencing of *PTGS2* in both TNBC and EOC cells resulted in decreased phosphorylation of STAT3 (Fig. S5D). TG101209, a potent inhibitor of JAK2/STAT3 signaling, suppresses STAT3 phosphorylation and thereby inhibits tumor cell growth 38. To further validate whether TN exerts its cytotoxic effect through inhibition of the JAK/STAT3 axis, we pretreated TNBC and EOC cells with TN for 12 h, followed by TG101209 treatment for 24 h. The CCK-8 assay indicated that pretreatment with TN attenuated the cytotoxicity induced by TG101209 (Fig. 5G). Similarly, pretreatment with TN significantly attenuated the cytotoxic effects of the STAT3 inhibitor STAT3-IN-3 in both TNBC and EOC cells (Fig. S5E). Subsequently, a rescue assay utilizing the STAT3 agonist ML115, in conjunction with CCK-8 viability assay, revealed that ML115 administration partially counteracted the cytotoxic effects of TN on both TNBC and EOC cells (Fig. 5H). To assess whether TN-mediated blockade of the JAK/STAT3 axis induce autophagy and apoptosis, western blot analysis and flow cytometry were performed. Western blot results demonstrated that ML115 treatment rescued the TN-induced downregulation of p62 and upregulation of LC3-II (Fig. 5I), while flow cytometry indicated that ML115 partially restored TN-induced apoptosis (Fig. 5J and Fig. S5F). Collectively, these results imply that TN degrades PTGS2 protein, thereby downregulating the STAT3/c-Myc pathway and inducing autophagic cell death, ultimately leading to the suppression of tumor cell growth (Fig. S5G).

### Triptonide suppresses TNBC and EOC tumorigenesis in xenograft and PDO models

**Figure 6:**
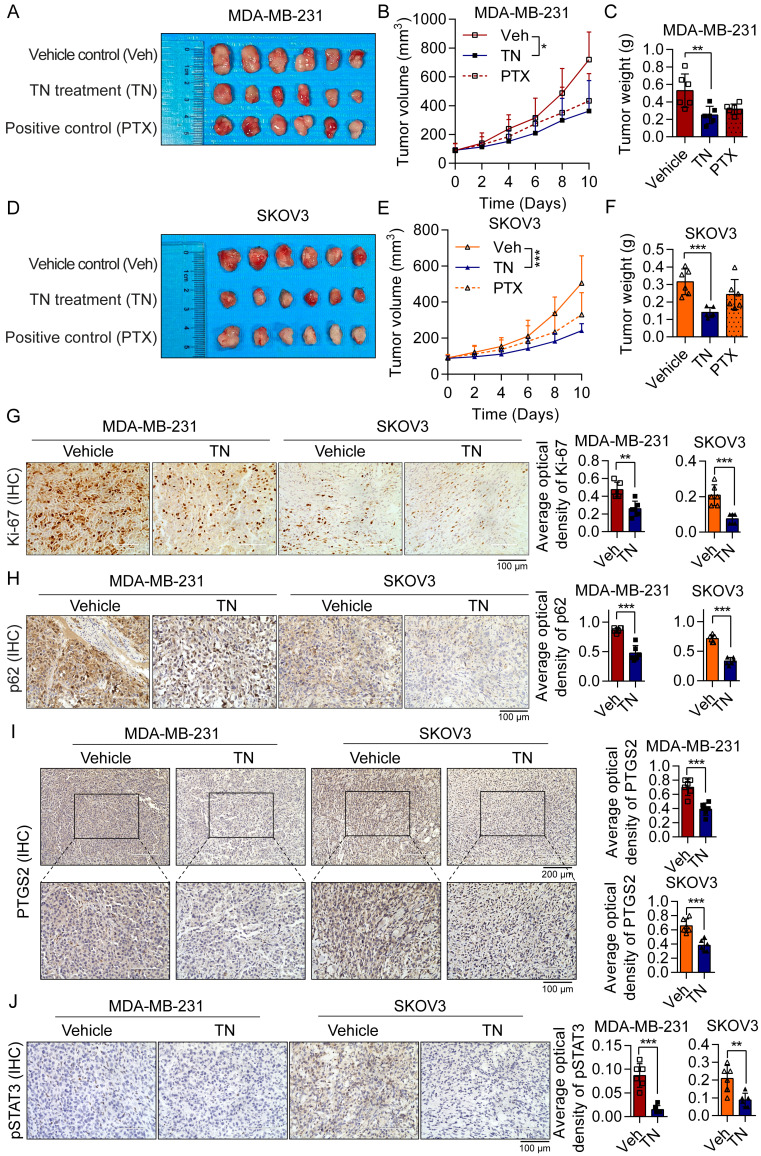
** Triptonide suppresses the growth of TNBC and EOC tumors *in vivo*. (A-C)** MDA-MB-231-derived xenograft mice divided into three groups (n = 6/group) were administered either the vehicle control, TN treatment, or positive control treatment. The resultant treatment effects were evaluated by tumor photographs (A), xenograft growth curves (B), and final tumor weights (C). **(D-F)** Three groups of SKOV3-derived xenograft mice (n = 6/group) were established and treated with vehicle control, TN, or positive control, respectively. The resultant treatment effects were evaluated by tumor photographs (D), xenograft growth curves (E), and final tumor weights (F). **(G-J)** IHC staining of Ki-67 (G), p62 (H), PTGS2 (I), and pSTAT3 (J) expression in vehicle-treated and TN-treated groups. Representative images (*left*) and quantitative analysis (*right*) are shown. Data are expressed as mean ± SD, with statistical significance assessed by one-way ANOVA, or Student's t-test; **P < 0.01, ***P < 0.001.

To evaluate the therapeutic efficacy of TN *in vivo*, we established xenograft models by subcutaneously injecting BALB/c nude mice with MDA-MB-231 cells (for TNBC) or SKOV3 cells (for EOC). The mice xenograft model results demonstrated that TN administration significantly inhibited tumor growth in both TNBC and EOC xenograft models, with tumor weight in the TN-treated groups being markedly reduced compared to the vehicle control groups (Fig. 6A-F). The IHC staining indicated that TN significantly suppressed the Ki-67 proliferation index of tumor xenografts (Fig. 6G), accompanied by a marked decrease in p62 expression (Fig. 6H). Moreover, in the TN-treated group, protein levels of PTGS2, phosphorylated STAT3, and c-Myc in tumor tissues were notably downregulated (Fig. 6I-J and Fig. S6A). These findings suggest that *in vivo* administration of TN suppresses the PTGS2/STAT3/c-Myc signaling axis and promotes autophagic cell death.

**Figure 7:**
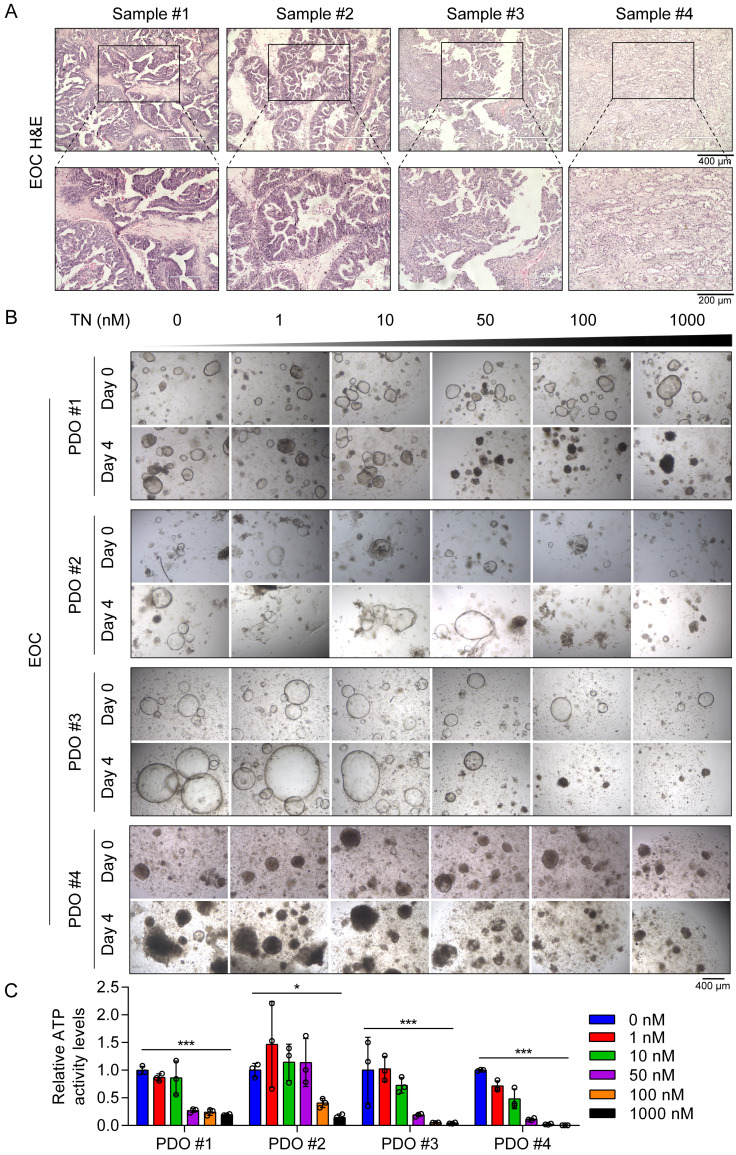
** Triptonide suppresses EOC tumorigenesis in PDO models. (A)** H&E staining of tumor tissue samples from patients with EOC used for PDO establishment. **(B-C)** PDOs treated with increasing concentrations of TN and cell viability measured on day 4 using an ATP-based assay. Representative images (B) and quantitative analysis of relative ATP activity levels in PDOs across treatment groups (C) are shown. Data are expressed as mean ± SD, with statistical significance assessed by one-way ANOVA; *P < 0.05, **P < 0.01, ***P < 0.001.

Furthermore, throughout the treatment period, body weight was monitored in all groups, and no significant differences were observed among the three groups (Fig. S6B). Additionally, H&E staining revealed no evident changes in the livers, spleens, or kidneys between the vehicle control group and the TN treatment group (Fig. S6C-E), indicating minimal systemic toxicity. To investigate the potential clinical application of TN in human tumor tissues, we established four EOC PDO models using surgical samples obtained from four patients with EOC (Fig. 7A). These PDOs were then treated with increasing concentrations of TN (0, 1, 10, 50, 100, and 1000 nM), and cell viability was assessed by measuring ATP levels. The results indicated that TN treatment markedly reduced both the size and number of PDOs in a concentration-dependent manner (Fig. 7B). Correspondingly, ATP levels were significantly diminished following TN exposure (Fig. 7C). We observed that PDO#1 and PDO#3, which showed heightened sensitivity to TN, exhibited elevated PTGS2 expression in their original tumor tissues (Fig. S7). Elevated PTGS2 expression is closely associated with enhanced proliferation and chemoresistance in various cancer cell types 39, 40. Here, the IC_50_ curve analyses revealed that TN significantly increased the sensitivity of TNBC and EOC cells to DDP (Fig. S8A). Furthermore, in two colorectal cancer cell lines, HCT116 and RKO, TN downregulated PTGS2 expression and demonstrated potent antitumor efficacy at nanomolar concentrations (Fig. S8B-C). Collectively, these results demonstrate that targeting PTGS2 with TN represents a promising therapeutic strategy for cancers exhibiting elevated PTGS2 expression.

## Discussion

Autophagy is a dynamically regulated cellular process that can play a dual role in tumor biology: it promotes cellular survival by clearing damaged components and maintaining homeostasis, and also facilitates cell death through excessive self-digestion and engagement of apoptotic pathways 41. In the present study, we demonstrated that TN significantly induces both autophagy and apoptosis in cancer cells. Although the precise mechanistic network requires further elucidation, our findings provide the preliminary evidence that TN induces autophagic cell death by directly binding to and promoting the degradation of PTGS2.

PTGS2 is a well-established mediator of inflammation and oncogene 42. The majority of PTGS2 inhibitors predominantly target inflammatory components such as cancer-associated fibroblasts and M2 macrophages within the tumor microenvironment, demonstrating broad-spectrum anti-inflammatory activity 43. However, these agents exhibit limited efficacy in directly suppressing PTGS2 expression within tumor cells, and this limitation is particularly significant given that PTGS2 is actively produced by cancer cells and contributes critically to cancer stemness, apoptotic resistance, and metastasis 44. Furthermore, certain chemotherapeutic agents inadvertently upregulate PTGS2 activity, underscoring the need for more selective therapeutic strategies that directly target tumor-specific PTGS2 overexpression 45. In this study, TN was identified as a novel inhibitor of PTGS2. Our results not only uncover a new mechanism through which TN exerts its anticancer effects but also highlight PTGS2 as a functionally relevant target in TN-treated TNBC and EOC models.

The molecular interactions between drugs and their target proteins, including hydrogen bonding, are critical for stabilizing drug-target complexes and enhancing pharmacological outcomes, and thus, play a fundamental role in determining binding affinity and therapeutic efficacy 46, 47. Our study revealed the His-207 residue of PTGS2 as a key site for TN binding, highlighting the importance of specific molecular interactions in mediating antitumor effects. The functional assay results demonstrated that mutation at His-207 abrogates the ability of TN to suppress tumor growth. This finding underscores the significance of residue-specific binding in PTGS2-targeted therapy and provides a structural insight into optimizing the mode of action of natural compounds derived from TCM. In addition, the transcriptomic data and western blot results reveal that *PTGS2* knockdown or TN treatment selectively suppresses the JAK/STAT3/c-Myc signaling axis, which is recognized as a central driver of oncogenesis, promoting tumor proliferation, metastasis, and therapy resistance 48, 49 in both TNBC and EOC cells. Our results elucidate a novel mechanism underlying the antitumor effects of TN, whereby TN binds to the PTGS2 protein at His-207, degrades it, and consequently blocks the pro-tumorigenic JAK/STAT3/c-Myc signaling cascade. Previous studies have demonstrated that TN effectively inhibits TNBC metastasis through the concurrent degradation of the Twist1 and Notch1 oncoproteins 24. In parallel, our findings revealed that treatment with STAT3 agonists or PTGS2 rescue failed to completely abrogate the tumor-suppressive effects of TN, indicating that TN functions as a multi-target degrader. In addition, tumor-derived prostaglandin E2 mediated by PTGS2 induces an immunosuppressive tumor microenvironment 50.

Thus, TN may act as a novel PTGS2 inhibitor to reverse this microenvironment and provide a new combination strategy for tumor immunotherapy. Furthermore, several current epi-drugs, including DNA-modifying agents, histone acetyltransferase inhibitors, and histone deacetylase inhibitors, have been used for breast cancer treatment 51. In the future, these drugs may also be used in combination with TN to reverse chemotherapy resistance and achieve better therapeutic effects. Drug delivery strategies are also an emerging research area. Sarkar et al. demonstrated that exosome-sheathed doxorubicin attenuates epithelial-mesenchymal transition in TNBC 52. This exosome-mediated drug delivery strategy may provide a novel paradigm for optimizing TN-targeted delivery.

Despite these promising results, this study has some limitations. The duration of TN treatment in our current xenograft experiments was limited to 10 days, which precluded us from investigating the effects of prolonged TN exposure on the systemic health status of mice and long-term tumor growth dynamics. For future investigations, we plan to administer TN subsequent to tumor cell injection to prolong its therapeutic duration. Furthermore, we will employ the MMTV-PyMT transgenic mouse model to evaluate the long-term impacts of TN treatment on tumor progression, metastasis, and the systemic homeostasis of the host. As TNBC is frequently diagnosed at advanced stages, access to treatment-naïve, surgically resected tissues is currently limited 10, thereby restricting the validation of TN sensitivity in patient-derived organoid models.

In conclusion, this study demonstrated that TN exerts significant antitumor effects in TNBC and EOC models, primarily through direct binding to the His-207 residue of PTGS2, leading to PTGS2 degradation with subsequent downregulation of the JAK/STAT3 signaling axis, ultimately inducing autophagic cell death. These results highlight PTGS2 as not only a prognostic biomarker but also a promising therapeutic target in these malignancies. Moreover, our work provides a compelling rationale for further development of TN as a potential anticancer agent.

## Supplementary Material

Supplementary figures and tables.

## Abbreviations

TNBC: triple-negative breast cancer
EOC: epithelial ovarian cancer
PTGS2: prostaglandin-endoperoxide synthase 2
TCM: traditional Chinese medicine
TN: triptonide
BIM: B cell lymphoma 2 interacting mediator of cell death
TMA: tumor tissue microarrays
DMEM: Dulbecco's modified Eagle medium
FBS: fetal bovine serum
CHQ: chloroquine
shRNA: short hairpin ribonucleic acid
siRNA: small interfering RNA
NEDD4: neural precursor expressed: developmentally down-regulated protein 4
SDS-PAGE: SDS-polyacrylamide gel electrophoresis
ACTB: β-Actin
GAPDH: glyceraldehyde-3-phosphate dehydrogenase
caspase-3: Cysteine-aspartic protease 3
ERK1/2: Extracellular signal-Regulated Kinase 1/2
STAT3: Signal Transducer and Activator of Transcription 3
TEM: transmission electron microscopy
qRT-PCR: quantitative reverse transcription polymerase chain reaction
SPR: surface plasmon resonance
CETSA: cell thermal shift assay
DARTs: drug affinity responsive target stability
GSEA: gene set enrichment analysis
H&E: hematoxylin and eosin
IHC: immunohistochemistry
PDO: patient-derived organoid
