# Supplementary material for: Triptonide-mediated PTGS2 Inhibition Induces Autophagic Cell Death to Suppress the Progression of Triple-negative Breast Cancer and Epithelial Ovarian Cancer
Source: Int J Biol Sci. 2026 Jan 30;22(5):2215–32. doi: 10.7150/ijbs.127562 (PMC12965061; doi:10.7150/ijbs.127562)
Supplement: Supplementary file 1 — Supplementary figures and tables. [file ijbsv22p2215s1.pdf]

Supplementary Information

Triptonide-mediated PTGS2 Inhibition Induces Autophagic Cell Death to Suppress the Progression of Triple-negative Breast Cancer and Epithelial Ovarian Cancer

Gong *et al.*

Scientific Research Center, First School of Clinical Medicine, The First Affiliated Hospital of Guangdong Pharmaceutical University, Guangzhou, 510080, China

*Correspondence:

Yinger Huang, E-mail: hyeyb@smu.edu.cn

Zhe-Sheng Chen, E-mail: chenz@stjohns.edu

Wenbo Hao, E-mail: haowa@126.com

Supplementary Figures

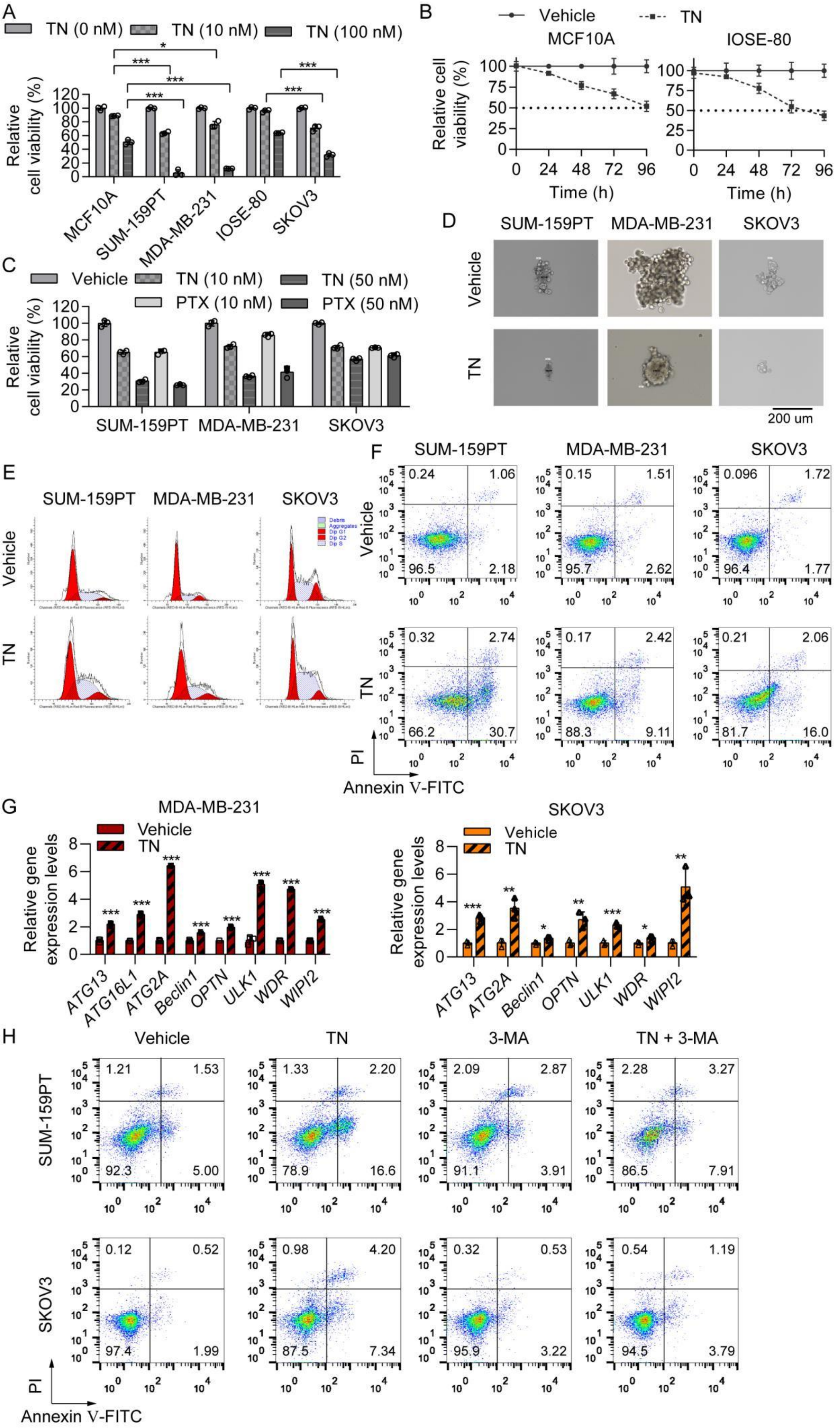
A
TN (0 nM)
TN (10 nM)
TN (100 nM)
Relative cell viability (%)
MCF10A
SUM-159PT
MDA-MB-231
IOSE-80
SKOV3
B
Vehicle
TN
MCF10A
IOSE-80
Relative cell viability (%)
Time (h)
C
Vehicle
TN (10 nM)
TN (50 nM)
PTX (10 nM)
PTX (50 nM)
Relative cell viability (%)
D
Vehicle
TN
200 um
E
F
0.24
1.06
96.5
2.18
0.15
1.51
95.7
2.62
0.096
1.72
96.4
1.77
0.32
2.74
66.2
30.7
0.17
2.42
88.3
9.11
0.21
2.06
81.7
16.0
PI
Annexin V-FITC
G
MDA-MB-231
SKOV3
Relative gene expression levels
ATG13
ATG16L1
ATG2A
Beclin1
OPTN
ULK1
WDR
WIPI2
H
3-MA
TN + 3-MA
1.21
1.53
92.3
5.00
1.33
2.20
78.9
16.6
2.09
2.87
91.1
3.91
2.28
3.27
86.5
7.91
0.12
0.52
97.4
1.99
0.98
4.20
87.5
7.34
0.32
0.53
95.9
3.22
0.54
1.19
94.5
3.79

18

Fig. S1. Triptonide significantly inhibits the proliferation and promotes apoptosis of TNBC and EOC cells.

(A) MCF10A, SUM-159PT, MDA-MB-231, IOSE-80 and SKOV3 cells were treated with varying concentrations of TN or vehicle, and cell proliferation was evaluated by a CCK-8 assay. **(B)** Growth curves of MCF10A and IOSE-80 cells treated with vehicle control or 40 nM TN at indicated time points, assessed by CCK-8 assay. **(C)** SUM-159PT, MDA-MB-231, and SKOV3 cells were exposed to different concentrations of TN or PTX for 48 h, and cell proliferation was assessed using a CCK-8 assay. **(D)** Tumorsphere formation assay of SUM-159PT, MDA-MB-231, and SKOV3 cells treated with 40 nM TN. Representative images are shown. **(E)** Cell cycle distribution analyzed by flow cytometry in SUM-159PT, MDA-MB-231, and SKOV3 cells after 24 h treatment with 40 nM TN. Representative histograms are displayed. **(F)** Apoptosis detected by flow cytometry in SUM-159PT, MDA-MB-231, and SKOV3 cells following 48 h exposure to 40 nM TN. Representative images are presented. **(G)** qPCR analysis of autophagy pathway-related gene expression in MDA-MB-231 and SKOV3 cells treated with TN for 24 h. **(H)** Apoptosis evaluated by flow cytometry in SUM-159PT and SKOV3 cells pretreated with 5 mM 3-MA for 6 h followed by 40 nM TN for 48 h. Representative diagrams are shown. Data are expressed as mean ± SD, with statistical significance assessed by Student's t-test; $*P < 0.05$, $**P < 0.01$, $***P < 0.001$.

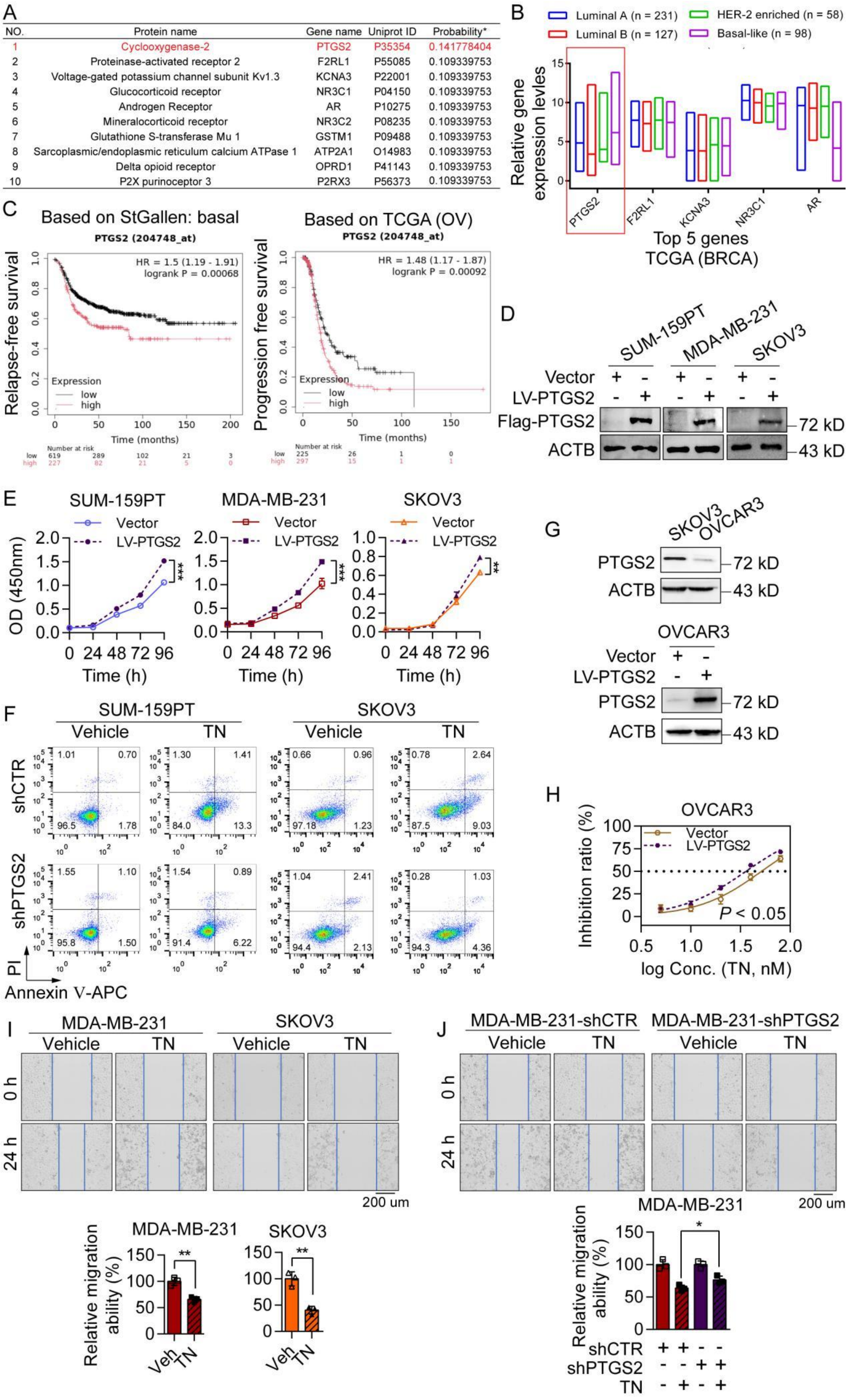
A
NO. Protein name Gene name Uniprot ID Probability*
1 Cyclooxygenase-2 PTGS2 P35354 0.141778404
2 Proteinase-activated receptor 2 F2RL1 P55085 0.109339753
3 Voltage-gated potassium channel subunit Kv1.3 KCNA3 P22001 0.109339753
4 Glucocorticoid receptor NR3C1 P04150 0.109339753
5 Androgen Receptor AR P10275 0.109339753
6 Mineralocorticoid receptor NR3C2 P08235 0.109339753
7 Glutathione S-transferase Mu 1 GSTM1 P09488 0.109339753
8 Sarcoplasmic/endoplasmic reticulum calcium ATPase 1 ATP2A1 O14983 0.109339753
9 Delta opioid receptor OPRD1 P41143 0.109339753
10 P2X purinoceptor 3 P2RX3 P56373 0.109339753
B
Luminal A (n = 231)
HER-2 enriched (n = 58)
Luminal B (n = 127)
Basal-like (n = 98)
Relative gene expression levles
PTGS2
F2RL1
KCNA3
NR3C1
AR
Top 5 genes
TCGA (BRCA)
C
Based on StGallen: basal
PTGS2 (204748_at)
HR = 1.5 (1.19 - 1.91)
logrank P = 0.00068
Relapse-free survival
Expression
low
high
Time (months)
Number at risk
Based on TCGA (OV)
PTGS2 (204748_at)
HR = 1.48 (1.17 - 1.87)
logrank P = 0.00092
Progression free survival
D
SUM-159PT
MDA-MB-231
SKOV3
Vector
LV-PTGS2
Flag-PTGS2
ACTB
72 kD
43 kD
E
SUM-159PT
MDA-MB-231
SKOV3
Vector
LV-PTGS2
OD (450nm)
Time (h)
F
SUM-159PT
SKOV3
Vehicle
TN
shCTR
shPTGS2
PI
Annexin V-APC
G
SKOV3
OVCAR3
PTGS2
ACTB
72 kD
43 kD
Vector
LV-PTGS2
H
OVCAR3
Vector
LV-PTGS2
Inhibition ratio (%)
P < 0.05
log Conc. (TN, nM)
I
MDA-MB-231
SKOV3
Vehicle
TN
0 h
24 h
200 um
Relative migration ability (%)
Veh
TN
J
MDA-MB-231-shCTR
MDA-MB-231-shPTGS2
Vehicle
TN
0 h
24 h
200 um
MDA-MB-231
Relative migration ability (%)
shCTR
shPTGS2
TN

Fig. S2. Triptonide targets the PTGS2 protein, which contributes to the progression of TNBC and EOC.

(A) Potential protein targets of TN. **(B)** mRNA expression levels of the top five potential targets of TN in breast cancer subtypes were analyzed based on the TCGA database. **(C)** Survival analysis of PTGS2 in patients with basal-like breast cancer (*top*) and EOC (*bottom*). **(D)** PTGS2 protein expression levels in vector control and PTGS2-overexpressing SUM-159PT, MDA-MB-231, and SKOV3 cells. **(E)** Growth rates of vector control and PTGS2-overexpressing SUM-159PT, MDA-MB-231, and SKOV3 cells were assessed by CCK-8 assay. **(F)** Apoptosis in TN-treated shRNA control and *PTGS2*-knockdown SUM-159PT and SKOV3 cells after 48 h was evaluated by flow cytometry. Representative diagrams are shown. **(G)** PTGS2 protein expression levels in SKOV3 and OVCAR3 cells (*top*) and in vector control and PTGS2-overexpressing OVCAR3 cells (*bottom*) were assessed by western blot analysis. **(H)** IC_{50} curves of TN in vector control versus PTGS2-overexpressing OVCAR3 cells. **(I)** Following 24 h treatment with 40 nM TN, changes in the migratory capacity of MDA-MB-231 and SKOV3 cells are shown as representative images (*top*) and corresponding statistical results (*bottom*). **(J)** The migratory ability of *PTGS2*-knockdown and shRNA control MDA-MB-231 cells after 24 h treatment with 40 nM TN. The representative images (*top*) and the corresponding statistical results (bottom) are shown. Data are reported as mean ± SD, with statistical significance evaluated via Spearman's correlation analysis, two-way ANOVA, or Student's t-test; $^{*}P < 0.05$, $^{**}P < 0.01$, $^{***}P < 0.001$.

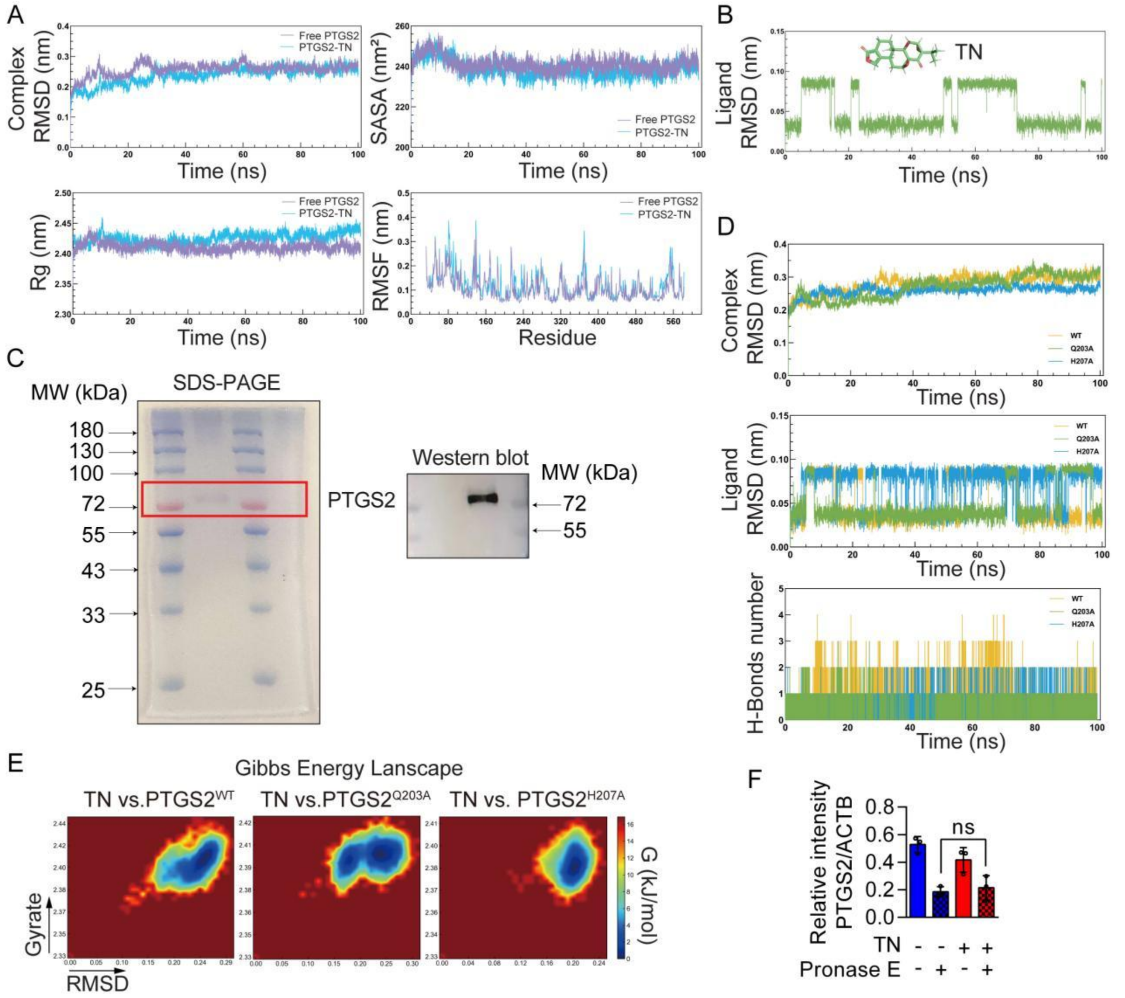

Fig. S3. Triptonide exerts anticancer bioactivity by directly binding to His-207 of the PTGS2 protein.

(A) Root mean square deviation (RMSD), radius of gyration (Rg), solvent accessible surface area (SASA), and root mean square fluctuation (RMSF) curves of free PTGS2 and PTGS2-TN complex. **(B)** Fluctuation trajectory of TN in the PTGS2-TN complex. **(C)** Purified PTGS2 protein detected by coomassie brilliant blue staining and western blot. **(D)** RMSD curves of PTGS2$^{WT/Q203A/H207A}$-TN systems, fluctuation trajectory of TN in PTGS2$^{WT/Q203A/H207A}$-TN complex, and hydrogen bond number variations in PTGS2$^{WT/Q203A/H207A}$-TN systems. **(E)** Two-dimensional Gibbs free energy landscape

of TN binding to PTGS2^{WT}, PTGS2^{Q203A}, and PTGS2^{H207A} proteins. **(F)** Degradation of PTGS2^{H207A} mutant protein in TN-treated cell lysate under pronase E treatment, detected by western blot. Quantitative analysis are shown. Data are expressed as mean ± SD, with statistical significance assessed by Student's t-test; ns, no significance.

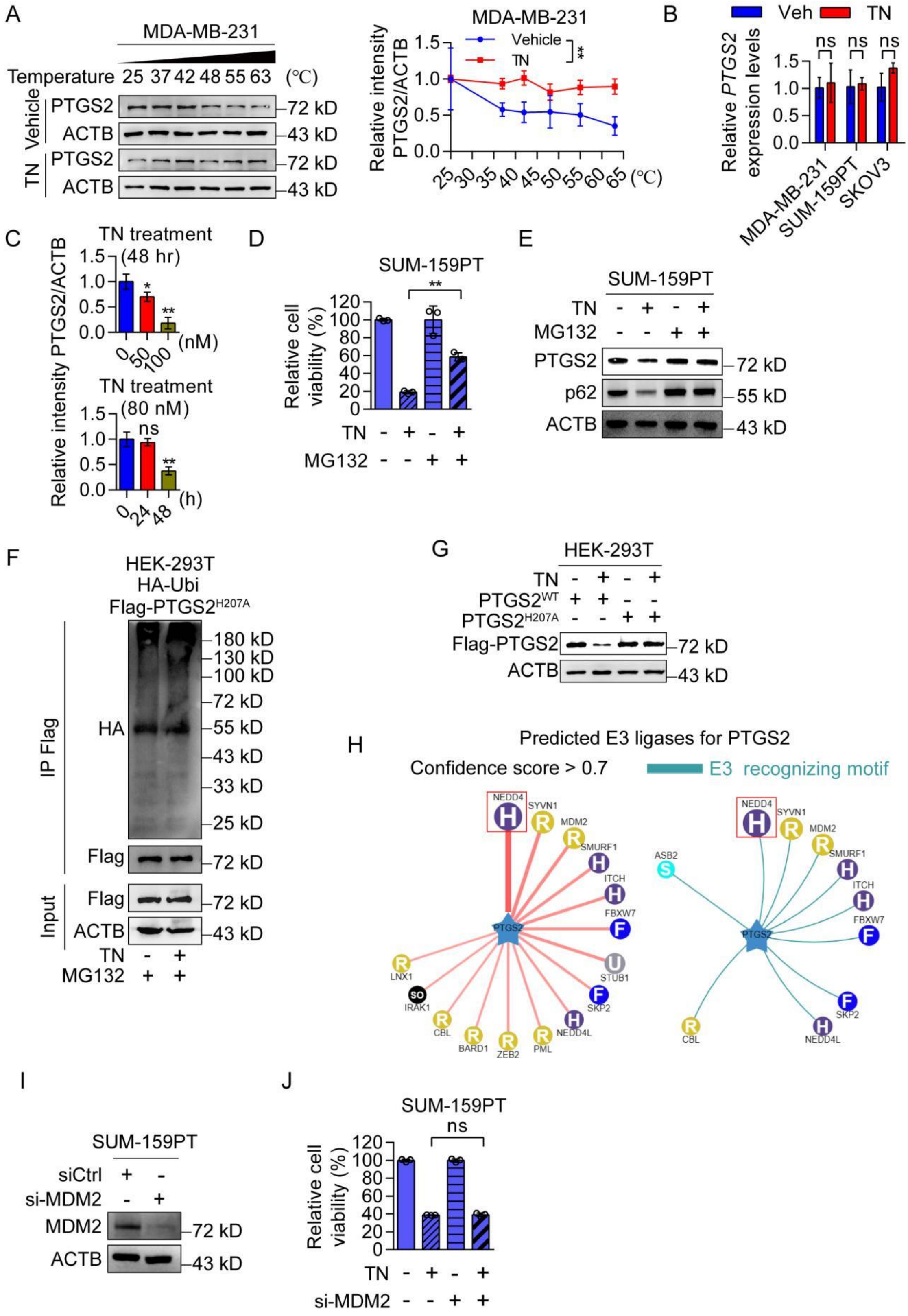

Fig. S4. Triptonide promotes PTGS2 degradation via the ubiquitin-proteasome system by recruiting the E3 ligase NEDD4.

(A) MDA-MB-231 cell lysate treated with TN was subjected to CETSA assay. Representative blot (*left*) and quantitative analysis (*right*) are shown. **(B)** qPCR analysis of *PTGS2* gene expression levels in TN-treated SUM-159PT, MDA-MB-231, and SKOV3 cells. **(C)** Western blot was performed to detect the evaluate the inhibitory effect of TN on PTGS2 protein expression. Quantitative analysis are shown. **(D)** SUM159-PT cells were pretreated with MG132 followed by 80 nM TN for 24 h, and cell viability was assessed using the CCK-8 assay. **(E)** SUM159-PT cells were pretreated with MG132 and then treated with 40 nM TN for 48 h. Protein expression levels of PTGS2 and p62 were detected by western blot. **(F)** HEK-293T cells co-expressing PTGS2^{H207A} and ubiquitin were pretreated with MG132, followed by treatment with 50 nM TN for 24 h, and the ubiquitination pattern of PTGS2^{H207A} protein was assessed by co-IP assay. **(G)** Western blot analysis was performed to evaluate the inhibitory effect of TN on wild-type and H207-mutated PTGS2 protein expression. **(H)** The E3 ubiquitin ligase mediating PTGS2 ubiquitination was predicted using UbiBrowser v2. **(I)** Western blot analysis of MDM2 knockdown efficiency. **(J)** CCK-8 assay was performed to assess the effect of MDM2 knockdown on TN-induced cytotoxicity. Data are reported as mean ± SD, with statistical significance evaluated via Spearman's correlation analysis, two-way ANOVA, or Student's t-test; $*P < 0.05$, $**P < 0.01$, ns, no significance.

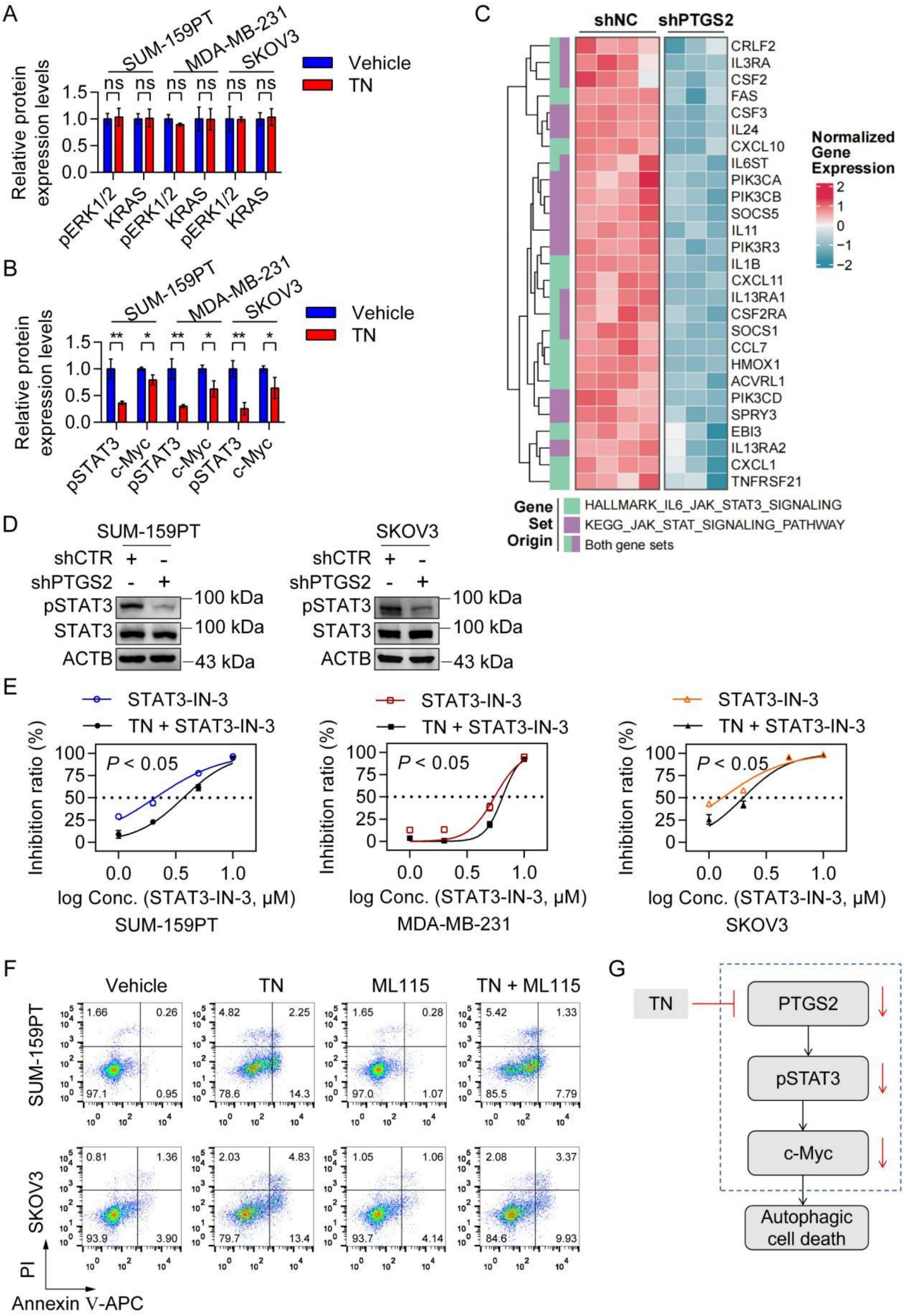

Fig. S5. Triptonide induces autophagic cell death by downregulating the JAK/STAT3 signaling pathway.

(A) Quantitative analysis of phosphorylated ERK and KRAS protein levels in SUM-

159PT, MDA-MB-231, and SKOV3 cells treated with TN. **(B)** Quantitative analysis of phosphorylated STAT3 and c-Myc protein levels in SUM-159PT, MDA-MB-231, and SKOV3 cells after TN treatment. **(C)** Expression of genes associated with Hallmark-IL6-JAK-STAT3 and KEGG-JAK-STAT signaling pathways in shRNA control and *PTGS2*-knockdown SUM-159PT cells. **(D)** Western blot analysis of pSTAT3 expression in SUM-159PT and SKOV3 cells transduced with shRNA control or PTGS2-targeting shRNA. **(E)** SUM-159PT, MDA-MB-231, and SKOV3 cells were pretreated with TN for 12 h, exposed to gradient concentrations of STAT-IN-3 for 24 h, and cell viability was assessed by CCK-8 assay. **(F)** Apoptosis detected by flow cytometry in SUM-159PT and SKOV3 cells treated with TN following ML115 administration. Representative diagrams are shown. **(G)** Schematic diagram of TN-mediated autophagic cell death in tumor cells. Data are reported as mean ± SD, with statistical significance evaluated via Spearman's correlation analysis, two-way ANOVA, or Student's t-test; *$P < 0.05$, **$P < 0.01$, ns, no significance.

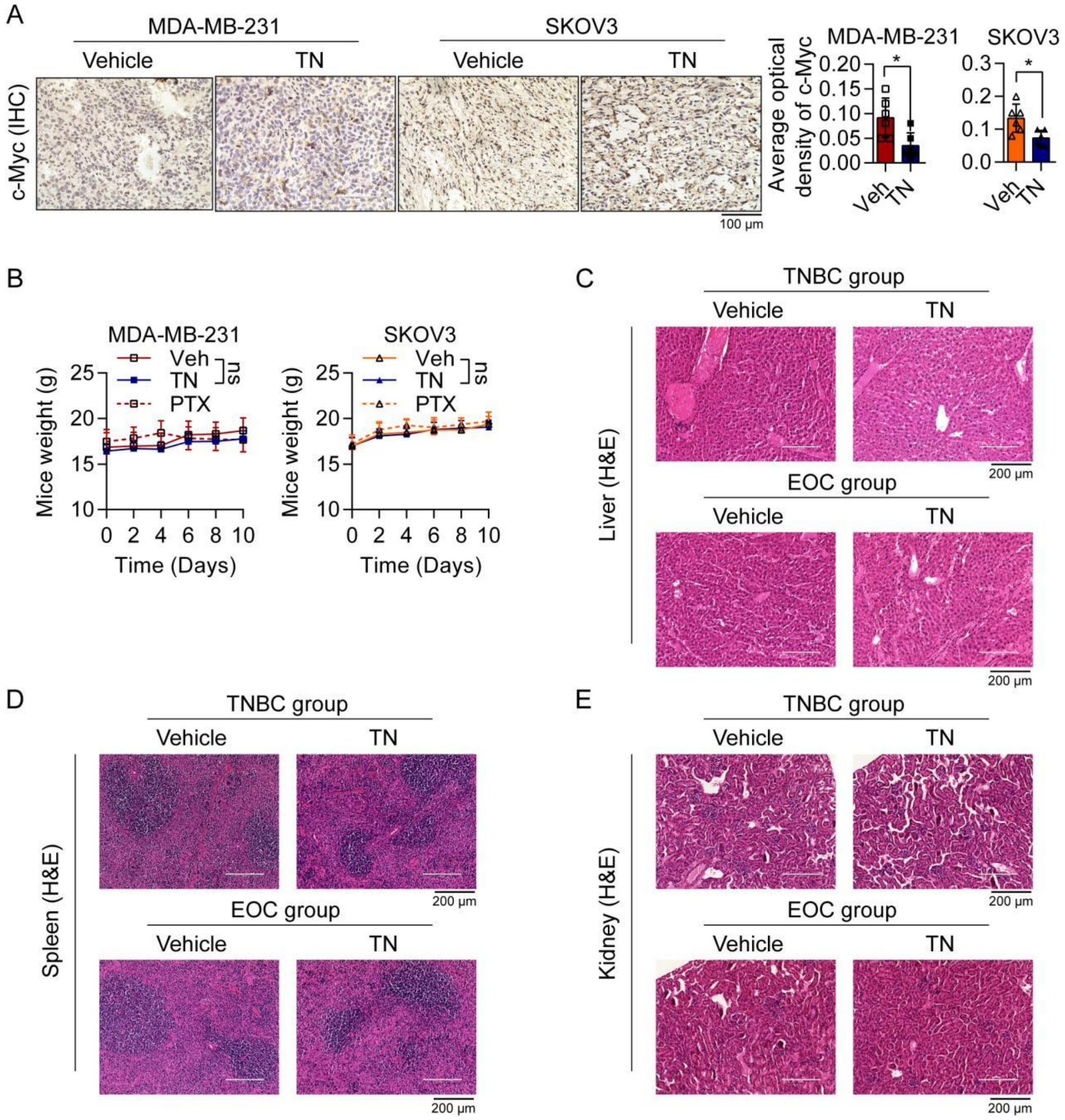

Fig. S6. Triptonide suppresses the growth of TNBC and EOC tumors *in vivo*.

(A) IHC staining of c-Myc expression in vehicle-treated and TN-treated groups. Representative images (*left*) and quantitative analysis are shown (*right*). **(B)** Body weight comparison across all experimental mouse groups. **(C-E)** H&E staining of important organs (liver, spleen, kidney) from vehicle-treated and TN-treated mice. Representative images are displayed for liver (C), spleen (D), and kidney (E). Data are reported as mean ± SD, with statistical significance evaluated via Spearman's correlation analysis, two-way ANOVA, or Student's t-test; **P* < 0.05, ns, no

significance.

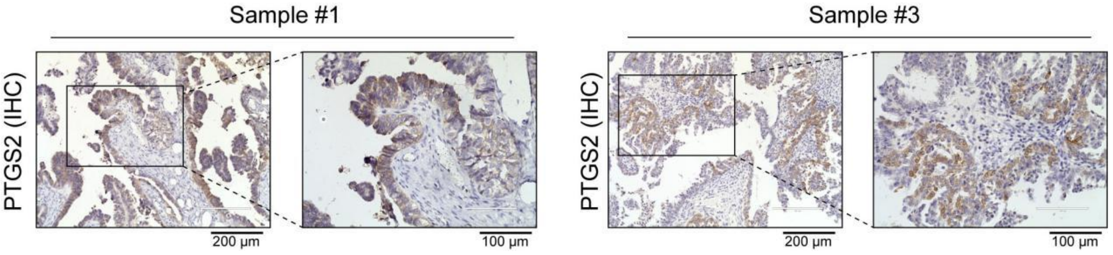

Fig. S7. Triptonide suppresses EOC tumorigenesis in PDO models.

IHC staining of PTGS2 expression in tumor tissue samples from patients with EOC, which were used for establishing PDO #1 and #3.

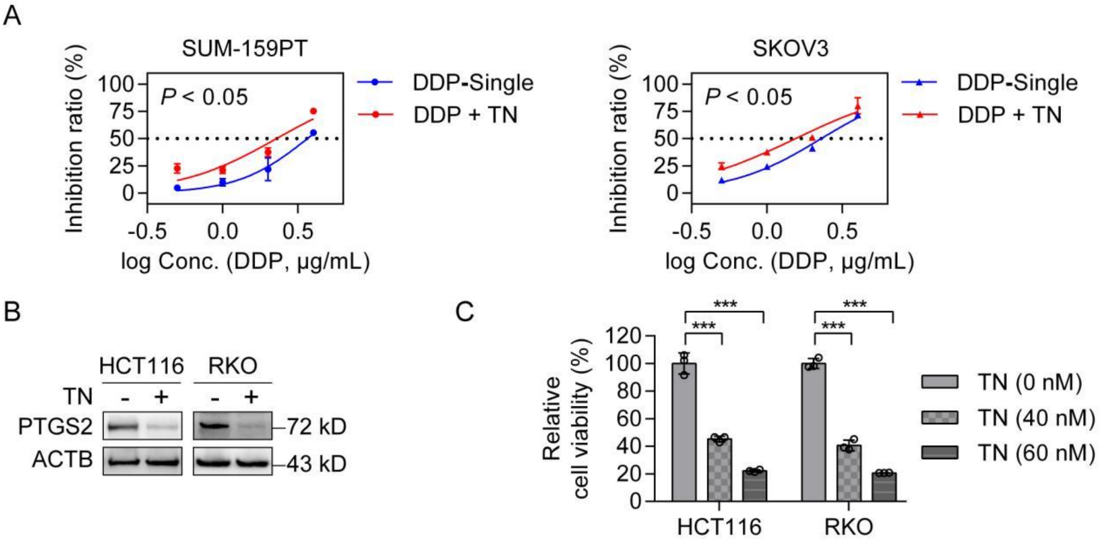

Fig. S8. TN-mediated chemosensitizing effect and cytotoxicity against colorectal cancer cells.

(A) IC_{50} curves for SUM159-PT and SKOV3 cells following treatment with DDP and/or TN (40 μM) for 48 h. **(B)** Western blot analysis of PTGS2 protein levels in HCT116 and RKO cells treated with DMSO or 40 nM TN for 48 h. **(C)** HCT116 and RKO cells were treated with varying concentrations of TN (0, 40, 60 nM) for 48 h,

and cell proliferation was evaluated by a CCK-8 assay. Data are reported as mean ± SD, with statistical significance evaluated via Spearman's correlation analysis, two-way ANOVA, or Student's t-test; ***$P < 0.001$.

Supplementary Tables

Table S1. Sequences used in this study.

Sequencse name	Sequence (5′-3′)
shCTR	CCGGTCCTAAGGTTAAGTCGCCCTCGCTCGAGCGAGGGCGACTTAACCTTAGGTTTTTGAATT
sh-*PTGS2*	CCGGCTATCACTTCAAACTGAAATTCTCGAGAATTTCAGTTTGAAGTGATAGTTTTTGAATT
siCtrl	UUCUCCGAACGUGUCACGUdTdT
si-*NEDD4*	CGUUCAGUCUCAAGAAAGATT
si-MDM2	CGCCACAAAUCUGAUAGUAUU

Table S2. Primers used in this study.

Gene	Forward Primers	Reverse Primers
GAPDH	5′ -TCTGACTTCAACAGCGACAC-3′	5′ -CGTTGTCATACCAGGAAATGAG-3′
ATG13	5′ -TCACTTTGTGGACCGTCCCTA-3′	5′ -TGGTACACACTTCTTGAGAGTCT-3′
ATG16L1	5′ -GGAGCTGGCCTGTGTTATGG-3′	5′ -GTGACATGTGGTCGGGAGAA-3′
ATG2A	5′ -TGTCCCTGTAGCCATGTTCG-3′	5′ -TCAGGATCTCCGTGTACTCAG-3′
Beclin 1	5’-AACCTTCCACATCTGGCACA-3’	5’-TCCGTAAGGAACAAGTCGGTA-3’

OPTN	5′ -CCAAACCTGGACACGTTTACC-3′	5′ -CCTCAAATCTCCCTTTCATGGC-3′
ULK1	5′ -GGCAAGTTCGAGTTCTCCCG-3′	5′ -CGACCTCCAAATCGTGCTTCT-3′
WDR45	5′ -GAGAAGCAACTGCTAGTGTTCC-3′	5′ -GGCTGGTTTAGAGACACACAG-3′
WIPI2	5′ -CCATCGTCAGCCTTAAAGCAC-3′	5′ -TCCAGGCATACTATCAGCCTC-3′
PTGS2	5′ -CTCCCTTGGGTGTCAAAGGTAAA-3′	5′ -AACTGATGCGTGAAGTGCTG-3′
MYC	5′ -GTAGTGGAAAACCAGCCTCCC-3′	5′ -CGTCGCAGTAGAAATACGGCT-3′
